# Kill and cure: genomic phylogeny and bioactivity of *Burkholderia gladioli* bacteria capable of pathogenic and beneficial lifestyles

**DOI:** 10.1099/mgen.0.000515

**Published:** 2021-01-18

**Authors:** Cerith Jones, Gordon Webster, Alex J. Mullins, Matthew Jenner, Matthew J. Bull, Yousef Dashti, Theodore Spilker, Julian Parkhill, Thomas R. Connor, John J. LiPuma, Gregory L. Challis, Eshwar Mahenthiralingam

**Affiliations:** ^1^​ Microbiomes, Microbes and Informatics Group, Organisms and Environment Division, School of Biosciences, Cardiff University, Cardiff, CF10 3AX, UK; ^2^​ Department of Chemistry and Warwick Integrative Synthetic Biology Centre, University of Warwick, CV4 7AL, UK; ^3^​ Warwick Integrative Synthetic Biology Centre, University of Warwick, Coventry CV4 7AL, UK; ^4^​ Department of Pediatrics, University of Michigan Medical School, Ann Arbor, Michigan, USA; ^5^​ Wellcome Trust Sanger Institute, Wellcome Trust Genome Campus, Hinxton, Cambridge CB10 1SA, UK; ^6^​ Department of Biochemistry and Molecular Biology, Biomedicine Discovery Institute, Monash University, Clayton, VIC 3800, Australia; ^†^​ Present address: School of Applied Sciences, Faculty of Computing, Engineering and Science, University of South Wales, Pontypridd, CF37 4BD, UK; ^‡^​ Present address: Pathogen Genomics Unit, Public Health Wales Microbiology Cardiff, University Hospital of Wales, Cardiff, CF14 4XW, UK; ^§^​ Present address: The Centre for Bacterial Cell Biology, Biosciences Institute, Medical School, Newcastle University, Newcastle upon Tyne, NE2 4AX, UK; ^#^​ Present address: Department of Veterinary Medicine, University of Cambridge, Madingley Road, Cambridge CB3 0ES, UK

**Keywords:** *Burkholderia*, *B. gladioli*, antibiotic production, plant pathogenesis, cystic fibrosis infection, phylogenomics

## Abstract

*
Burkholderia gladioli* is a bacterium with a broad ecology spanning disease in humans, animals and plants, but also encompassing multiple beneficial interactions. It is a plant pathogen, a toxin-producing food-poisoning agent, and causes lung infections in people with cystic fibrosis (CF). Contrasting beneficial traits include antifungal production exploited by insects to protect their eggs, plant protective abilities and antibiotic biosynthesis. We explored the genomic diversity and specialized metabolic potential of 206 *B. gladioli* strains, phylogenomically defining 5 clades. Historical disease pathovars (pv.) *B. gladioli* pv. *allicola* and *B. gladioli* pv. *cocovenenans* were distinct, while *B. gladioli* pv. *gladioli* and *B. gladioli* pv. *agaricicola* were indistinguishable; soft-rot disease and CF infection were conserved across all pathovars. Biosynthetic gene clusters (BGCs) for toxoflavin, caryoynencin and enacyloxin were dispersed across *B. gladioli *, but bongkrekic acid and gladiolin production were clade-specific. Strikingly, 13 % of CF infection strains characterized were bongkrekic acid-positive, uniquely linking this food-poisoning toxin to this aspect of *B. gladioli* disease. Mapping the population biology and metabolite production of *B. gladioli* has shed light on its diverse ecology, and by demonstrating that the antibiotic trimethoprim suppresses bongkrekic acid production, a potential therapeutic strategy to minimize poisoning risk in CF has been identified.

## Data Summary

*Burkholderia gladioli* genome sequences determined as part of this study are deposited under BioProjects PRJEB35318 and PRJEB9765. Complete genomes are available for 4 strains (BCC1622, GCA_900608515; BCC1621, GCA_900608525; BCC1710, GCA_900608535; BCC0238, GCA_900631635.1) and 206 draft genomes were determined with the accession numbers provided in Table S1 (available in the online version of this article).

**Impact Statement**

*
Burkholderia gladioli* is a fascinating bacterium with detrimental traits of plant pathogenicity, food-poisoning toxicity and the ability to cause lung infections in people with cystic fibrosis (CF). In contrast, because *B. gladioli* can produce multiple antimicrobial specialized metabolites, it can protect insects and plants, and has been recently exploited as an antibiotic producer. To understand the genomic and metabolic basis of this diversity, we genome-sequenced a collection of 206 *B. gladioli* isolates, and examined the specialized metabolites they encode and produce. While all agriculturally defined *B. gladioli* disease pathovars (pv.) were found to represent a single species, *B. gladioli* pv. *allicola* and *B. gladioli* pv. *cocovenenans* were evolutionarily distinct. In addition, the latter food-poisoning strains were unified as a group because they all encoded the potent toxin bongkrekic acid. Worryingly, 13 % of *B. gladioli* recovered from CF lung infection also encoded the toxin and could produce bongkrekic acid when grown in conditions mimicking CF sputum. Although identification of toxin-positive strains represents a potential new risk factor for CF, we showed that the clinically used antibiotic trimethoprim was able to suppress toxin bongkrekic acid production. Our genomic and metabolic analysis of *B. gladioli* has broad impacts for agriculture, biotechnology, chemistry and medicine.

## Introduction

The genus *Burkholderia* contains important plant, animal and human pathogenic bacteria [1, 2], as well as environmentally beneficial species [3]. Recently, amino acid- and nucleotide-based analyses have split *Burkholderia* strains into distinct lineages corresponding to *Burkholderia sensu stricto*, *Paraburkholderia *, *Caballeronia *, *Robbsia *, *Trinickia* and *Mycetohabitans* [4]. Within *Burkholderia sensu stricto*, the *Burkholderia cepacia* complex group of species are problematic lung pathogens in people with cystic fibrosis (CF) [2]. The three most commonly isolated *Burkholderia* species among US CF patients are *B. multivorans *, *B. cenocepacia* and, interestingly, *B. gladioli* [2]. Although phenotypically similar, genetically *B. gladioli* is not a member of the *B. cepacia* complex, but is part of a group of species associated with plant disease, including *B. glumae* and *B. plantarii* [3]. In relation to CF infection, *B. gladioli* may cause severe systemic abscesses [5] and is also considered a risk factor for lung transplantation, since it is associated with poor clinical outcome [6]. While the potential for patient-to-patient spread and rapid clinical decline are identified traits of *B. cepacia* complex infection in people with CF [2], the population biology, epidemiology and genomics of *B. gladioli* as a lung pathogen are essentially unknown.

In relation to its environmental ecology, *B. gladioli* was originally isolated as a pathogen of the flowering plant genus *Gladiolus* and its taxonomy has been updated several times [7]. The current species encompasses the historical *Gladiolus* disease causing taxa *Pseudomonas gladioli* and *Pseudomonas marginata* [8], the food poisoning-associated *B. cocovenenans* [7]*,* and the potential biological control agent *Pseudomonas antimicrobica* [9]. *B. gladioli* has also been isolated as a pathogen of important crops that resulted in pathovar (pv.) designations being applied to the causative isolates of: mushroom rot, *B. gladioli* pv. *agaricicola* [10]; onion rot, *B. gladioli* pv. *allicola* [11]; and the historical bulb rot disease, *B. gladioli* pv. *gladioli* [8]. *B. gladioli* and its close relative *B. glumae* are also major rice pathogens causing panicle blight [12]. *B. cocovenenans* represents a fourth pathovar [13] that is responsible for food poisoning when tempe bongkrek, the fermented coconut-based Indonesian national dish, is produced with *Rhizopus* fungal cultures contaminated with *B. gladioli* [14]. Under these conditions, a polyketide biosynthetic gene cluster (BGC) is activated in *B. gladioli* pv. *cocovenenans* that directs the production of the respiratory toxin bongkrekic acid, which is fatal when ingested [14]. The *B. gladioli* pathovars had been assigned based on the source of isolates and researchers have argued that there is a need to differentiate the lethal toxin-producing pathovars such as *B. gladioli* pv. *cocovenenans* [13]. However, the evolutionary basis of the pathovar designations of *B. gladioli* remains to be systematically investigated.

The capacity to produce a diverse range of specialized metabolites ranging from toxins such as bongkrekic acid [14] to beneficial antibiotics is a common trait among *Burkholderia* bacteria [1, 15]. Close ecological associations with multiple eukaryotic hosts is a key primer for metabolite production by *B. gladioli *. As a detrimental trait, it produces toxoflavin a yellow phytotoxin that enhances the virulence of *B. gladioli* in rice disease [16]. In addition to bongkrekic acid, *B. gladioli* produces the polyketide antibiotic enacyloxin IIa in co-culture with the fungus *Rhizopus microspores* [17]. A close association of *B. gladioli* with fungi was linked to the discovery that the bacterium contains a gene encoding a nonribosomal peptide synthetase (NRPS) that assembles icosalide A1, a metabolite originally characterized as a product of an *Aureobasidium* fungus [18, 19]. PCR screening of DNA extracts from the original *Aureobasidium* culture demonstrated that a *B. gladioli* symbiont containing the icosalide NRPS gene was present [19]. The vertical transmission of symbiotic *B. gladioli* in herbivorous *Lagriinae* beetles clearly demonstrates how ecological benefit may derive from the metabolites the bacterium produces [20]. *B. gladioli* was found in the reproductive tract of the beetles and produced several antimicrobial metabolites, including toxoflavin, caryoynencin, lagriene (*iso*-gladiolin [21]) and sinapigladioside, which protected the *Lagriinae* eggs from fungal attack [20].

Genomics has revolutionized our understanding of *Burkholderia* population biology, and the beneficial and detrimental interactions of these ecologically diverse bacteria. Insights into the biosynthesis of gladiolin, a novel polyketide antibiotic with promising activity against *Mycobacterium tuberculosis *, were facilitated by complete genome sequencing of *B. gladioli* BCC0238 [21]. Using a combination of systematic approaches, including genome mining for specialized metabolite BGCs, metabolite characterization and phenotypic assays, production of the antimicrobial cepacin was shown to underpin biological control of damping-off disease by the biopesticide species *Burkholderia ambifaria* [22]. However, a limited number of complete genome sequences are available for *B. gladioli *, including strain BSR3, a rice disease isolate [23], the bulb-associated type strain ATCC 10248 [24], and the CF lung infection isolate, BCC0238 [21]. Here we investigate the population biology of *B. gladioli* as a functionally diverse species that interacts with human, plant, insect and microbial ecosystems. Using genome sequence analysis of 206 *B. gladioli* strains from diverse sources, we defined the genetic linkage to pathovar status, mapped the ability to mediate plant soft-rot and human disease, and correlated population biology to capacity for specialized metabolite production. The genomics-based taxonomy of all the *B. gladioli* isolates was consistent with their designation as a single species. Pathovars *B. gladioli* pv. *allicola* and *B. gladioli* pv. *cocovenenans,* as well as BGCs for bongkrekic acid and gladiolin, were shown to be clade-restricted within the overall *B. gladioli* population. People with CF were susceptible to all clades of *B. gladioli* and the presence of the bongkrekic acid BGC was revealed as a new risk factor for these infectious isolates.

## Methods

### Bacterial strains and growth conditions

A collection of 206 *B. gladioli* isolates was assembled for this study and their source details, genomic features and the analysis they were subject to are included in the Supplementary Material (Table S1). These were drawn from the Cardiff collection [21, 22, 25] and the *Burkholderia cepacia* Research Laboratory and Repository (University of Michigan, MI, USA) [2], with additional reference and pathovar strains of *B. gladioli* obtained from the Belgium Coordinated Collection of Microorganisms (Ghent, Belgium) and National Collections of Plant Pathogenic Bacteria (York, UK) (Table S1). *B. gladioli* isolates were routinely grown on tryptone soya agar (TSA) or in tryptone soya broth (TSB) liquid cultures, and incubated at 37 °C. Antibiotic production was induced by growing strains on a minimal salts medium with glycerol as the sole carbon source (designated basal salts medium with glycerol; BSM-G) as previously described [25, 26].

Antimicrobial antagonism assays were performed by overlaying with the following susceptibility testing organisms: *Staphylococcus aureus* ATCC 25923, *Ralstonia mannitolilytica* LMG 6866 and *Candida albicans* SC 5314, as previously described [25]. *Escherichia coli* strain NCTC 12241 was used as a control for the mushroom and onion rot assays. Artificial CF sputum medium was made up as previously described [27] to model whether bongkrekic acid production occurred under CF lung infection-like growth conditions. Trimethoprim (1 µg ml^−1^) was incorporated into BSM-G to determine whether *B. gladioli* metabolite production was induced by sub-inhibitory concentrations of this antibiotic, as described for *Burkholderia thailandensis* [28].

### Genome sequencing, assembly and analysis

Genomic DNA was prepared from 3 ml TSB overnight cultures of *B. gladioli *. Cells were harvested by centrifugation and suspended in 400 µl of 4 M guanidine isothiocyanate solution (Invitrogen, UK). DNA was extracted from these bacterial suspensions using a Maxwell 16 automated nucleic acid purification system and the Maxwell tissue DNA purification kit following the manufacturer’s instructions (Promega, UK). Purified DNA extracts were treated with RNase (New England BioLabs, UK). Genomes were sequenced using the Illumina HiSeq 2000 and HiSeq X Ten platforms at the Wellcome Sanger Institute as previously described [22]. Genomes were assembled from the read data, and annotated and compared using a virtual machine hosted by the Cloud Infrastructure for Microbial Bioinformatics (CLIMB) consortium [29]. Sequence reads were trimmed using Trim Galore v0.4.2 (Babraham Bioinformatics), overlapped using FLASH v1.2.11 [30], and assembled using SPAdes v3.9.1 [31]. Assembled genomes were polished using Pilon v1.21 [32]. Prokka v1.12 beta [33] was used for gene prediction and annotation. The quality of genome assemblies was assessed using Quast and Prokka annotations cross-compared with gene predictions generated by Glimmer v3.02b [34]. Draft genome contigs were ordered against a complete reference genome for *B. gladioli* BCC0238 [21] using CONTUGuator v2.7 [35]. To supplement the Illumina sequencing, contiguated genomes were generated for clade-specific strains BCC1710, BCC1621 and BCC1622 (see Table S1) using Pacific Biosciences single-molecule real-time sequencing as described previously [21].

Average nucleotide identity (ANI) was used for genomic taxonomy and calculated using PyANI v0.2.1 [36]. The *B. gladioli* core genome was computed using Roary v3.6.0 [37]. Maximum-likelihood trees were drawn from the core gene alignment with FastTree [38] using the generalized time-reversible model of nucleotide evolution and visualized using FigTree (http://tree.bio.ed.ac.uk/software/figtree). Rooting the trees with multiple *Burkholderiales* species (* Burkholderia glumae *, *Burkholderia oklahomiensis* and *Paraburkholderia xenovorans *) failed to produce a biologically meaningful root. The closest sequences to these outgroups were variable, but all produced trees of consistent phylogenetic separation for the *B. gladioli* clades identified. Therefore, an unrooted tree was presented in the final analysis. *B. gladioli* multilocus sequencing typing (MLST) sequence type (ST) assignments were made by using the PubMLST database and website [39], via the MLST tool developed by Torsten Seemann (https://github.com/tseemann/mlst). Putative specialized metabolite BGCs in *B. gladioli* genomes were identified using antiSMASH v3 [40, 41] running as a local instance on CLIMB. The presence or absence of known BGCs was determined by mapping sequencing reads to representative BCG reference sequences using snippy (https://github.com/tseemann/snippy). The percentage of reads mapping to the reference sequence, and the actual number of corresponding reads were used to manually determine the status of each BGC in a given strain.

### Mushroom and onion rot bioassays

Mushroom (*Agaricus bisporus*) soft-rot bioassays were carried out as described previously [42] with the surface sterilization and immersion into ice-cold water step omitted because this caused non-specific rotting of mushrooms. Briefly, mushrooms (Oakland closed cup mushroom, Lidl UK GmbH, produced in Ireland) were cut into 3–4 mm slices with a sterile blade. *B. gladioli* was grown overnight in TSB and the cap of each mushroom was inoculated with a 10 µl drop of bacterial suspension adjusted to 0.1 OD_600 nm_ in TSB. Onion (*Allium cepa*) soft-rot bioassays were carried out as described previously [43]. Brown onions (Tesco, Cardiff, UK) had their skin and outer onion layer removed prior to quartering with a sterile knife. Individual onion layers were cut into 3 to 4 cm pieces and wounded on their inner surface with a knife slit made under aseptic conditions, and the wound was inoculated with 10 µl of bacterial suspension produced as described for the mushroom assay. All assays (the test *B. gladioli *, a control *E. coli* NCTC 12241 strain, a TSB control and untreated controls) were performed in triplicate on sterile wet filter paper contained in sterile 9 cm plastic Petri dishes, sealed with Parafilm M and incubated at 30 °C for 48 h.

### Preparation of *B. gladioli* metabolite extracts and antimicrobial activity

To analyse the metabolites produced by different *B. gladioli* strains, BSM-G agar plates (five per strain) were streaked with cells from a freshly revived culture and incubated for 72 h at 30 °C. Biomass was removed using a sterile cell scraper and the spent agar was transferred to a glass bottle. Metabolites were extracted from the agar using dichloromethane (2 h with gentle shaking). The crude extract was concentrated to dryness under a vacuum at 22 °C and resuspended in 1 ml of dichloromethane. The bioactivity of each extract and control dichloromethane was tested by pipetting 5 µl onto a TSA plate and allowing the plates to dry and solvent to evaporate. Each plate was then overlaid with molten Iso-Sensitest agar (Oxoid, UK) seeded with *S. aureus *, *R. mannitolilytica* or *C. albicans* as described elsewhere [25]. Plates were incubated at 37 °C for 24 h and photographed to document zones of clearing. Bioactivity assays were performed in triplicate for each strain.

### Analysis of *B. gladioli* metabolites by high-performance liquid chromatography (HPLC)

HPLC analysis was used to quantify and identify known *B. gladioli* metabolites as follows. Specialized metabolites were produced by growing on BSM-G medium (as above) and extracted directly from a 20 mm agar disc cut from the plates as described elsewhere [44]. Extracts (20 µl injection volume) were analysed on a Waters AutoPurification HPLC System fitted with a reverse-phase analytical column (Waters XSelect CSH C18, 4.6×100 mm, 5 µm) and a C18 SecurityGuard cartridge (Phenomenex) in series. Absorbance at 210–400 nm was monitored using a photo diode array (PDA) detector. Mobile phases consisted of (a) water with 0.1 % formic acid and (b) acetonitrile with 0.1 % formic acid. A flow rate of 1.5 ml min^−1^ was used. Elution conditions were as follows. 0–1 min: 95 % (a)/5 % (b); 1–9 min: gradient of (a) from 95–5 %/gradient of (b) from 5–95 %; 10–11 min: 5 % (a)/95 % (b); 11–15 min: 95 % (a)/5 % (b). Peak height and area were calculated using MassLynx V4.1 software (www.waters.com).

To enable BGC-specialized metabolite correlations, a *B. gladioli* gene mutant unable to produced gladiolin [21] was used. *B. gladioli* toxoflavin, bongkrekic acid and caryoynencin non-producing mutants were constructed as follows. PCR products encoding fragments of core biosynthetic genes were amplified using specific primers (see Table S2) and cloned into the pGpΩTp suicide plasmid [45] following digestion with *Xba*I/*Eco*RI (*bonA* and *cayA*), or *Xba*I/*Kpn*I (*toxA*). Plasmids were mobilized as described elsewhere[21] into *B. gladioli* BCC0238 to inactivate the gladiolin and toxoflavin BGCs, strain BCC1710 for the bongkrekic acid BGC and strain BCC1697 to disrupt the caryoynencin BGC. Comparative HPLC analysis of metabolite extracts from parental and mutant strains, combined with high-resolution liquid chromatography mass spectrometry (LC-MS) analysis (see below), was used to identify BGC products.

### High-resolution LC-MS of *B. gladioli* metabolite production

Known *Burkholderia* metabolites were confirmed by high-resolution LC-MS essentially as described elsewhere [19, 21, 22, 25]. Briefly, all *B. gladioli* strains were grown at 30 °C on BSM-G agar. Single plates were extracted by removal of the biomass, chopping of the agar and extraction with 4 ml of ethyl acetate for 2 h. Centrifugation in a 1.5 ml Eppendorf tube was used to remove debris. Crude extracts were analysed directly by Ultra-high performance liquid chromatography coupled with electrospray ionization-quadrupole-time of flight-mass spectrometry (UHPLC-ESI-Q-TOF-MS) analyses using a Dionex UltiMate 3000 UHPLC connected to a Zorbax Eclipse Plus C-18 column (100×2.1 mm, 1.8 µm) coupled to a Bruker MaXis II mass spectrometer. Mobile phases consisted of water (a) and acetonitrile (b), each supplemented with 0.1 % formic acid. A gradient of 5 % (b) to 100 % (b) over 30 min was used at a flow rate of 0.2 ml min^−1^. The mass spectrometer was operated in either positive or negative ion mode with a scan range of 50–3000 *m/z*. Source conditions were: end-plate offset at −500 V, capillary at −4500 V, nebulizer gas (N_2_) at 1.6 bar, dry gas (N_2_) at 8 l min^−1^ and dry temperature at 180 °C. Ion transfer conditions were: ion funnel radio frequency (RF) at 200 Vpp, multiple RF at 200 Vpp, quadrupole low mass at 55 *m/z*, collision energy at 5.0 eV, collision RF at 600 Vpp, ion cooler RF at 50–350 Vpp, transfer time at 121 μs and pre-pulse storage time at 1 μs. Calibration was performed with 1 mM sodium formate through a loop injection of 20 µl at the start of each run.

To analyse bongkrekic acid production in artificial CF sputum medium [27], 50 ml cultures of *B. gladioli* were grown for 3 days and centrifuged to remove bacterial cells, after which the supernatant was passed through a 2 µm filter to remove all cells and sterilize. The filtrate then passed through Sep-Pak C18 Vac Cartridge (500 mg), washed with two column volumes of water and finally eluted with 6 ml of methanol. The methanol was concentrated to 500 µl for bongkrekic acid detection by high-resolution LC-MS as above.

### PCR detection of the bongkrekic acid BGC

To detect the presence of the bongkrekic acid BGC, PCR probes were designed to target the central polyketide synthase gene, *bonA*, in the gene cluster from *B. gladioli* BCC1710 (*bonA*-F, 5′-ATTTCTAGAAGTATCCGCATTTTCGTCGC-3′; *bonA*-R 5′-TATGAATTCGATCGATCAGTTGCGCTTCC-3′). PCRs were performed using the *Taq* PCR Core kit (Qiagen) as per the manufacturer’s instructions and incorporating Q-solution.

The thermal cycling conditions comprised an annealing temperature of 54.5 °C and an extension time of 1 min 5 s, run over 30 cycles. The 1053 bp amplicon internal to *bonA* was detected by gel electrophoresis. The amplicon from the BCC1710 strain was subjected to Sanger sequencing (Eurofins, Genomics) to confirm its identity.

### Accession numbers

The sequence read data from the *B. gladioli* isolates examined in this study are available from the European Nucleotide Archive under the project accession numbers PRJEB9765 and PRJEB35318 [46]; isolate accession numbers are provided in Table S1.

## Results

### Assembly and genomic taxonomy of a *B. gladioli* isolate collection

To provide a holistic understanding of taxonomy and pathovar population biology of *B. gladioli *, a representative collection of 206 isolates was assembled and their genomes sequenced (Table S1). The majority of isolates (*n*=194) were from people with CF, with 181 from the USA, 7 from the UK, 4 from Canada, and 1 each from Australia and Italy (Table S1). Twelve strains were from environmental sources including pathovar reference isolates as follows: isolates of plant disease-associated *B. gladioli* pv. *gladioli* (*n*=3), pv. *agaricicola* (*n*=3) and pv. *alliicola* (*n*=3), and *B. gladioli* pv. *cocovenenans* (*n*=2) toxin-producing strains. One *B. gladioli* isolated from an environmental industrial source was also included (BCC1317; Table S1). Short-read sequencing yielded high-quality draft genome sequences [average of 82 contigs, ranging from 20 (BCC1721) to 284 (BCC1788)] with a mean size for *B. gladioli* of 8.28 Mb. The mean GC content was 68 % and the genomes contained a mean of 6872 protein-encoding genes (Table 1). These metrics were consistent with previously reported *B. gladioli* genomes [21, 23, 24].

**Table 1.** Summary genome sequencing statistics (n=206)

	Mean	Maximum	Minimum
*No. of contigs*	82.6	284 (BCC1788)	20 (BCC1721)
*Genome size*	8.28 Mb	8.94 Mb (BCC1815)	7.32 Mb (BCC1681)
*N50*	404 821	1 964 774 (BCC1691)	147 146 (BCC1713)
*%GC*	68.0 %	68.31 % (BCC1823)	67.39 % (BCC1815)
*No. of genes*	6872	7693	6012

Since the assignment of isolates within the genus *Burkholderia* [4] generally, and *B. gladioli* specifically [7, 9], has undergone multiple rounds of taxonomic reclassification, we initially established whether the 206 *B. gladioli* isolates in the collection comprised a single bacterial species. Using average nucleotide identity, the 96.85 % ANI for the entire *B. gladioli* 206 genome dataset was above the 95 % cut-off used in genomic taxonomy for designation as a single species [47]. This confirmed that the previous incorporation of *B. cocovenenans* (strains LMG 11626 and LMG 18113) [7] and *P. marginata* (ATCC10248) [8] into *B. gladioli* is supported by the genomic taxonomy (Table S1) [47].

**Fig. 1.**
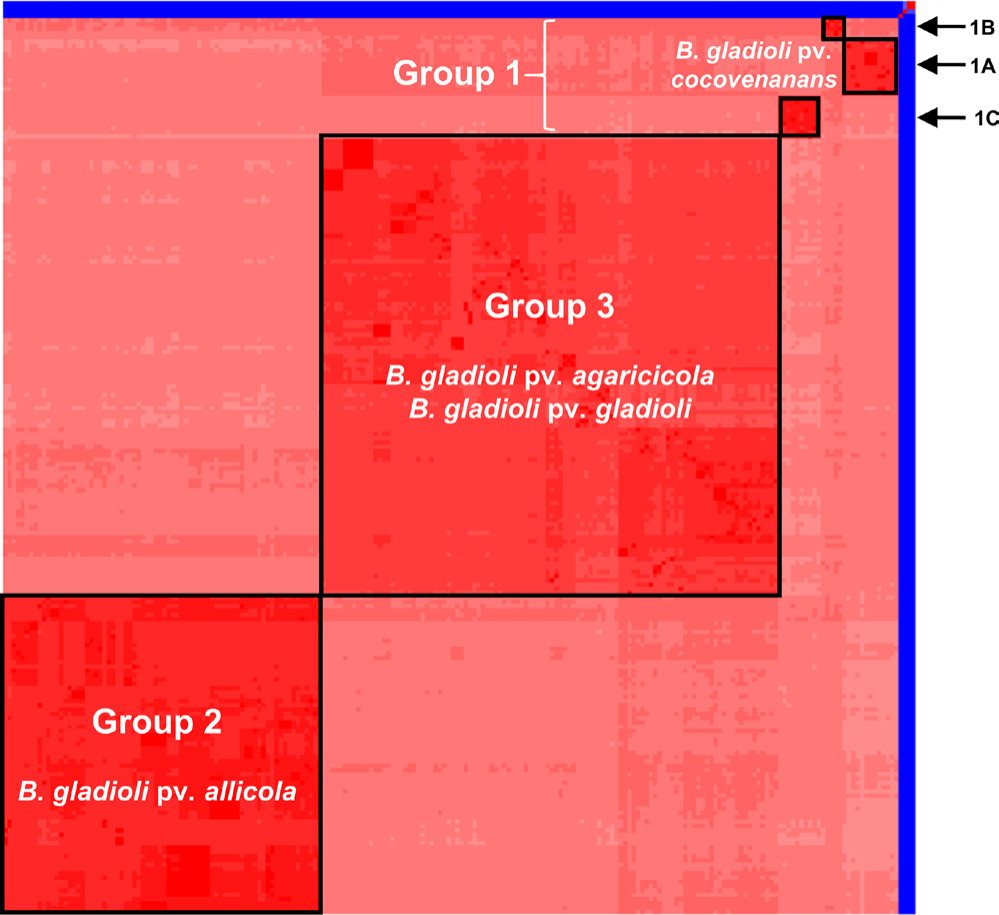
*
B. gladioli* comprises a single genomic species with evidence of subspecies clustering by average nucleotide identity. The ANI of 206 *B. gladioli* genomes was compared using pyANI and a heatmap was constructed (see the Methods section). Darker red shading correlates to the greater percentage identity of each isolate. Sub-groups with greater than 98.8 % ANI are shown within the black outlined boxes. The main clusters are labelled as group 1, 2 and 3, with group 1 isolates sub-dividing further into sub-groups 1A, 1B and 1C (see top right). Four non-* B. gladioli* species genomes were used as taxonomic controls: three strains of the closely related species *B. glumae* and one strain of *B. ambifaria *; their low nucleotide identity to *B. gladioli* (ANI <95 %) is shown in blue.

ANI heatmap analysis also suggested that a significant subspecies population structure existed within *B. gladioli* (Fig. 1), and as such the following designation of groups was made. Group 1 (*n*=27) comprised three closely related sub-groups: 1A, containing the reference *B. gladioli* pv. *cocovenenans* strains, 1B and 1C; each subgroup was distinct in terms of their ANI relatedness (Fig. 1). All isolates within each 1A, 1B and 1C subgroup contained the bongkrekic acid BGC (see below; Table S1), setting them apart from the rest of the *B. gladioli* collection, and supporting their collective designation as group 1. Group 2 was composed of 73 strains and included all 3 *B. gladioli* pv. *allicola* reference isolates. Group 3 (*n*=106) contained both the *B. gladioli* pv. *agaricicola* and *B. gladioli pv. gladioli* reference isolates (Fig. 1). Within each of these three initial groupings, the genomic ANI ranged from >98.1 % (group 3) to >99.1 % (group 1B), which was greater than the 96.85 % collection average and suggested that distinct genetic lineages were present within *B. gladioli* (Fig. 1).

### Core gene phylogenomic analysis reveals distinct evolutionary clades within *B. gladioli*

**Fig. 2.**
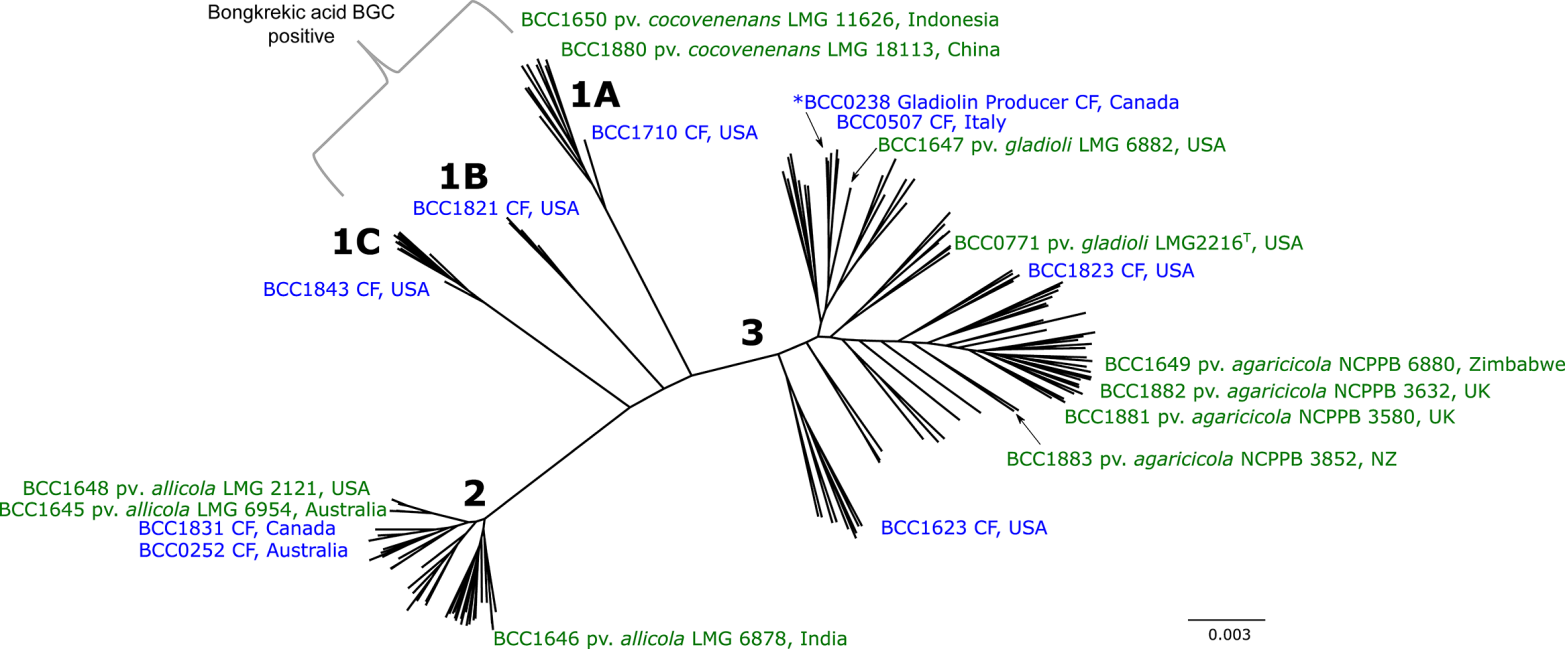
The population biology of *B. gladioli* inferred by core genome gene phylogeny. The core genome of the 206 *B. gladioli* strains were analysed using Roary and the resulting 4406 core genes were aligned and used to construct a phylogeny. A maximum-likelihood unrooted tree was constructed using the generalized time-reversible model of nucleotide evolution; multiple rooting with species outside of *B. gladioli* was used to confirm topology (see the Methods section). The approximate positions of the environmental *B. gladioli* pathovar reference strains are shown (green font), together with representative CF isolates (purple font) and the country of origin for each isolate. The position of the model gladiolin-producing strain, BCC0238, is indicated by * (see group 3). The five major evolutionary branches consistent with the ANI groups and sub-groups are numbered accordingly. Clades 1A,1B and 1C all contained the bongkrekic acid BGC and are collectively designated as group 1 *B. gladioli* (see brackets). The scale bar represents the number of base substitutions per site.

To investigate the evolutionary linkages behind the ANI groupings (Fig. 1), we constructed a phylogeny from the 4406 core genes identified within the 206 *B. gladioli* genome dataset. The strain groups defined by ANI (Fig. 1) were also supported as distinct clades in the phylogenomic analysis (Fig. 2). The three group 1 ANI sub-clusters correspondingly separated as clades 1A, 1B and 1C, with the reference *B. gladioli* pv. *cocovenenans* strains locating specifically to clade 1A (Fig. 2). These group 1 strains separated as 13 isolates in clade 1A, 4 in clade 1B and 10 in clade 1C (Table S1). At the distal ends of the *B. gladioli* phylogenetic tree were clade 2 and clade 3 strains (Fig. 2), which corresponds to the respective ANI groupings (Fig. 1). All three reference *B. gladioli* pv. *allicola* strains mapped to clade 2, indicating that the pathovar status is evolutionarily supported. However, *B. gladioli* pv. *agaricicola* and pv. *gladioli* grouped within clade 3 and were not genetically distinguished (except that they were distinct from clades 1 and 2; Fig. 2). Core gene content did not vary extensively across the phylogenomic lineages, ranging from 4682 for group 2 to 5109 for group 3 *B. gladioli *, with the group 1 genomes encoding 4847 core genes (Table S3). The significance of core gene differences between the lineage remains to be determined, but a defining conserved feature of the group 1 genomes was the presence of the bongkrekic acid BGC.

### Ecological and disease associations of *B. gladioli* evolutionary clades

**Fig. 3.**
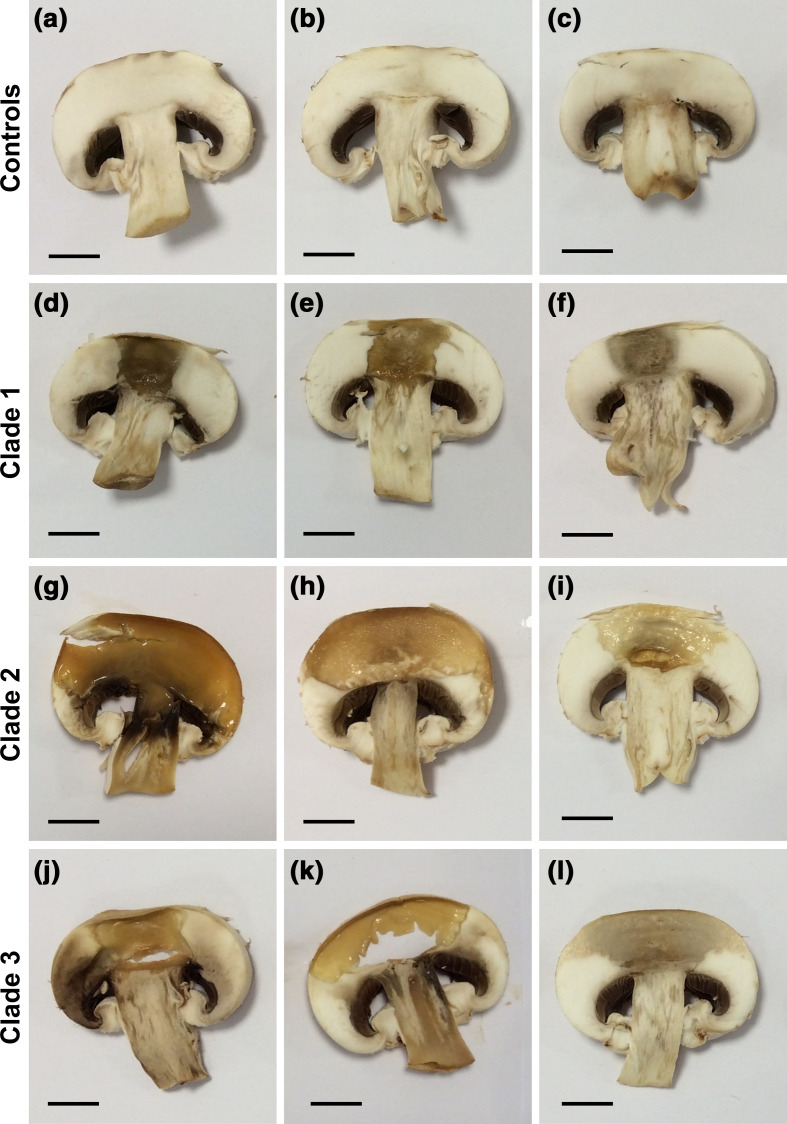
*
B. gladioli* from all evolutionary clades are capable of mushroom rot. The ability of *B. gladioli* strains to degrade mushroom tissue was tested by inoculating commercial mushroom tissue slices with standardized bacterial cultures. Inoculated mushrooms were incubated at 30 °C for 48 h with the following shown in each panel (each row are either controls or *B. gladioli* clades as indicated on the left): (a) no-treatment control, (b) TSB only, (c) *E. coli* NCTC 12241, (d) *B. gladioli* clade 1A strain BCC1710, (e) clade 1B strain BCC1675, (f) clade 1C strain BCC1678, (g) *B. gladioli* clade 2 strain BCC1731, (h) *B. gladioli* pv*. allicola* reference clade 2 strain BCC1645, (i) B. *gladioli* pv*. allicola* reference clade 2 strain BCC1646, (j) *B. gladioli* clade 3 strain BCC0238, (k) *B. gladioli* pv*. agaricola* reference clade 3 strain BCC1883 (NCPPB 3852), (l) *B. gladioli* pv*. gladioli* reference clade 3 strain BCC771 (LMG 2216^T^). Pitting and tissue degradation was apparent in all *B. gladioli *-inoculated mushrooms; a scale bar (1 cm) is shown in each panel to enable comparison.

Given the evolutionary support for the clade restriction of *B. gladioli* pv. *allicola*, and grouping of pv. *gladioli* and pv. *agaricicola* strains in a separate clade (Fig. 2), the ability of selected *B. gladioli* strains to rot eukaryotic tissues was investigated. Mushroom soft-rot bioassays [42] demonstrated that *B. gladioli* strains from all three phylogenomic groups were capable of decaying mushroom tissue (Fig. 3). The assay confirmed the ability of the pv. *agaricicola* reference strain NCPPB 3852 (BCC1883; Fig. 3k) to cause disease in its originally associated host. The degree of mushroom rot observed varied, with severe degradation of the mushroom cap tissue most apparent in clade 2 and 3 strains, compared with clade 1 producing less extensive rot (Fig. 3). The pathovar *agaricicola-*like strains therefore did not appear to be specifically adapted to degrade mushroom tissue. *B. gladioli* from all three clades also showed conserved plant tissue degradation capabilities within an onion soft-rot model [43]. A variable onion rot phenotype was observed for each strain, with the most extensive tissue pitting seen in clades 2 and clade 3 (Fig. S1). Overall, rotting capability was demonstrated by the *B. gladioli* strains from all genetic groups.

Since 94 % of the 206 *B. gladioli* strain collection derived from CF lung infections (Table S1), this disease source was also the major origin for each of the clades, demonstrating that opportunistic human pathogenicity was also a shared species phenotype (Fig. 2). For the 181 CF strains originating from the USA, mapping the state location of the submitting CF treatment centre showed that *B. gladioli* infections were geographically widespread with no phylogeographic linkages to clade types (Fig. S2). The group 1 *B. gladioli* strains (Figs 1 and 2), which possess the ability to produce bongkrekic acid (see below), were also found to be capable of causing CF lung infections, linking them to opportunistic lung disease for the first time.

### *B. gladioli* possess broad antimicrobial bioactivity

**Fig. 4.**
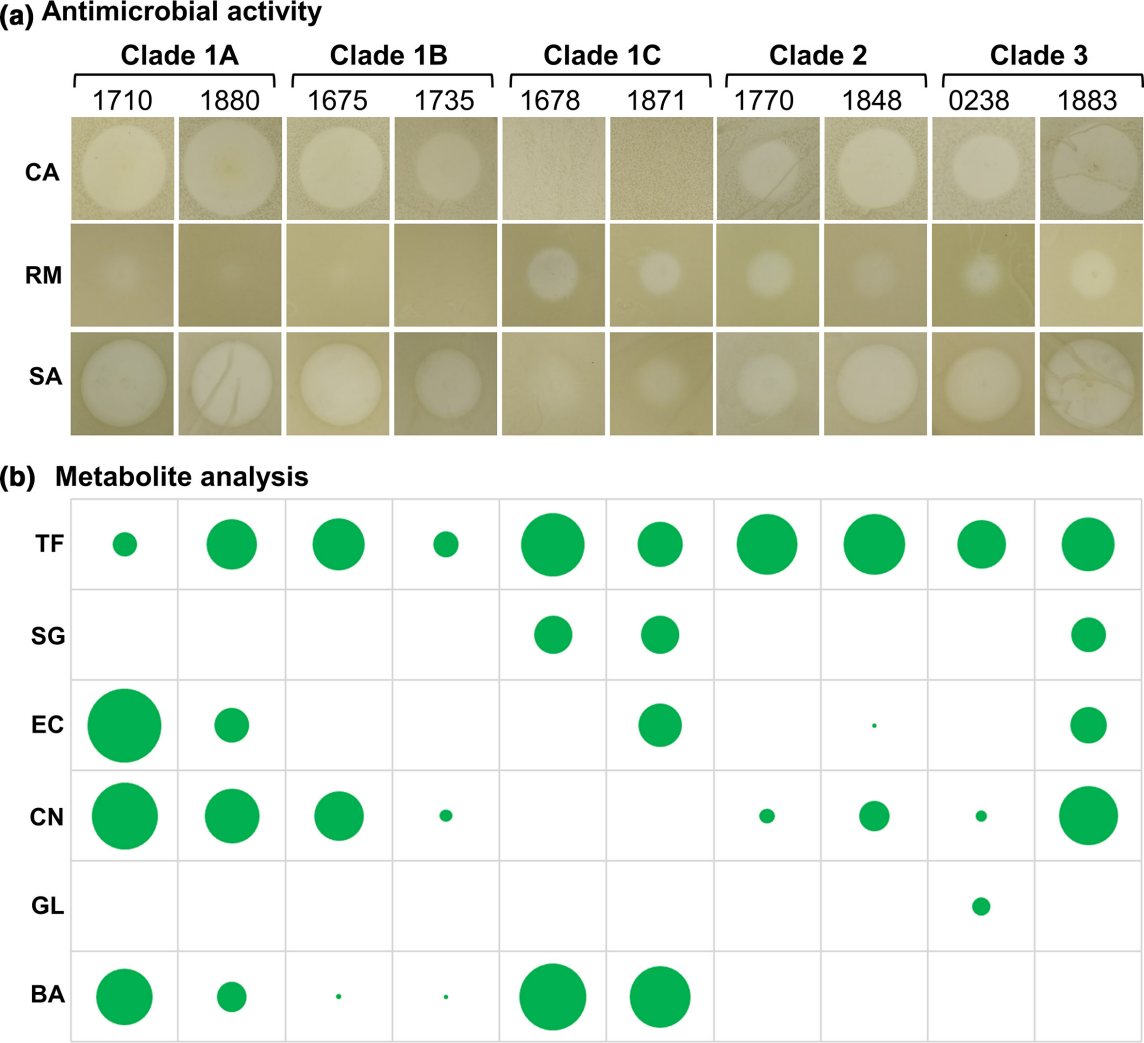
*
B. gladioli* antimicrobial activity and bioactive metabolite analysis. (a) The bioactivity of metabolites extracts made from spent growth media from *B. gladioli* cultures. The bioactivity from equivalent metabolite extracts of strains representative of each clade are shown as follows (clade and BCC number are labelled): *B. gladioli* Clade 1A strains BCC1710 and BCC1880, Clade 1B strains BCC1675 and BCC1735, and Clade 1C strains BCC1678 and 1871; *B. gladioli* Clade 2 strains BCC1770 and BCC1848 and *B. gladioli* Clade 3 strains BCC0238 and *B. gladioli pv. agaricicola* reference Clade 3 strain BCC1883 (NCPPB 3852). Each area of bioactivity was cropped to scale and represents a 3 cm section of the inoculated petri dish. Zones of growth inhibition against *C. albicans* (CA), *R. mannitolilytica* (RM) and *S. aureus* (SA) are labelled as rows. (b) The quantitative analysis of known *B. gladioli* metabolites present in the metabolite extracts as determined by HPLC. Each circle is proportionally scaled to the mean peak height for the following metabolites: toxoflavin (TF), sinapigladioside (SG), enacyloxin IIa (EC), caryoynencin (CN), gladiolin (GL) and bongkrekic acid (BA).

*B. gladioli* is known to produce an array of bioactive specialized metabolites, including toxoflavin [16], bongkrekic acid [14], enacyloxins [17], caryoynencin [48], sinapigladioside [20], gladiolin [21] and icosalides [18, 19]. Given this wealth of bioactive products, two *B. gladioli* strains representative of each clade within the species population biology (Fig. 2) were screened for antimicrobial activity. Metabolite extracts from the spent agar of *B. gladioli* cultures were examined for activity against Gram-positive and Gram-negative bacteria, and fungi. All 10 strains tested demonstrated activity against *S. aureus *. Only the two isolates from *B. gladioli* clade 1C lacked antifungal activity, while the extracts from the *B. gladioli* clade 1C, clade 2 and clade 3 strains possessed activity against Gram-negative bacteria (Fig. 4a). Overall, this analysis demonstrated that all the *B. gladioli* strains secreted extractable bioactive compounds, but the quantity and spectrum of activity varies (Fig. 4a).

To determine which metabolites accounted for the *B. gladioli* bioactivity (Fig. 4b), a combination of HPLC, high-resolution LC-MS (Fig. S3) and BGC pathway mutagenesis was employed. Under growth conditions that are known to promote specialized metabolite biosynthesis [25], toxoflavin was produced by all *B. gladioli* strains tested (Figs 4b and S3a). The isothiocyanate sinapigladioside [20] was produced by both clade 1C strains and one clade 3 strain (Fig. S3f). Enacyloxin IIa [25] was present in both clade 1A strains, a clade 1C strain and a clade 3 strain, and was also detected at low quantities within the clade 2 strain BCC1848 (Figs 4b and S3b). Production of the polyyne caryoynencin [48] (Fig. S3g) was widespread. It was detected in 8 of the 10 strains tested, and was only absent from clade 1C strains (Fig. 4b). Gladiolin [21] was detected in the *B. gladioli* BCC0238 strain it was originally discovered from (Fig. S3e), but was absent from the other strains examined (Fig. 4b). Bongkrekic acid (Fig. S3c) was detected in all six clade 1 strains, although only limited amounts were present in the two clade 1B strains examined (Fig. 4b). Overall, the metabolite analysis showed that individual *B. gladioli* strains were capable of producing up to four different bioactive metabolites (Fig. 4b), underpinning the broad-spectrum antimicrobial activity of *B. gladioli* (Fig. 4a).

### Distribution of known specialized metabolite BGCs in *B. gladioli*

**Fig. 5.**
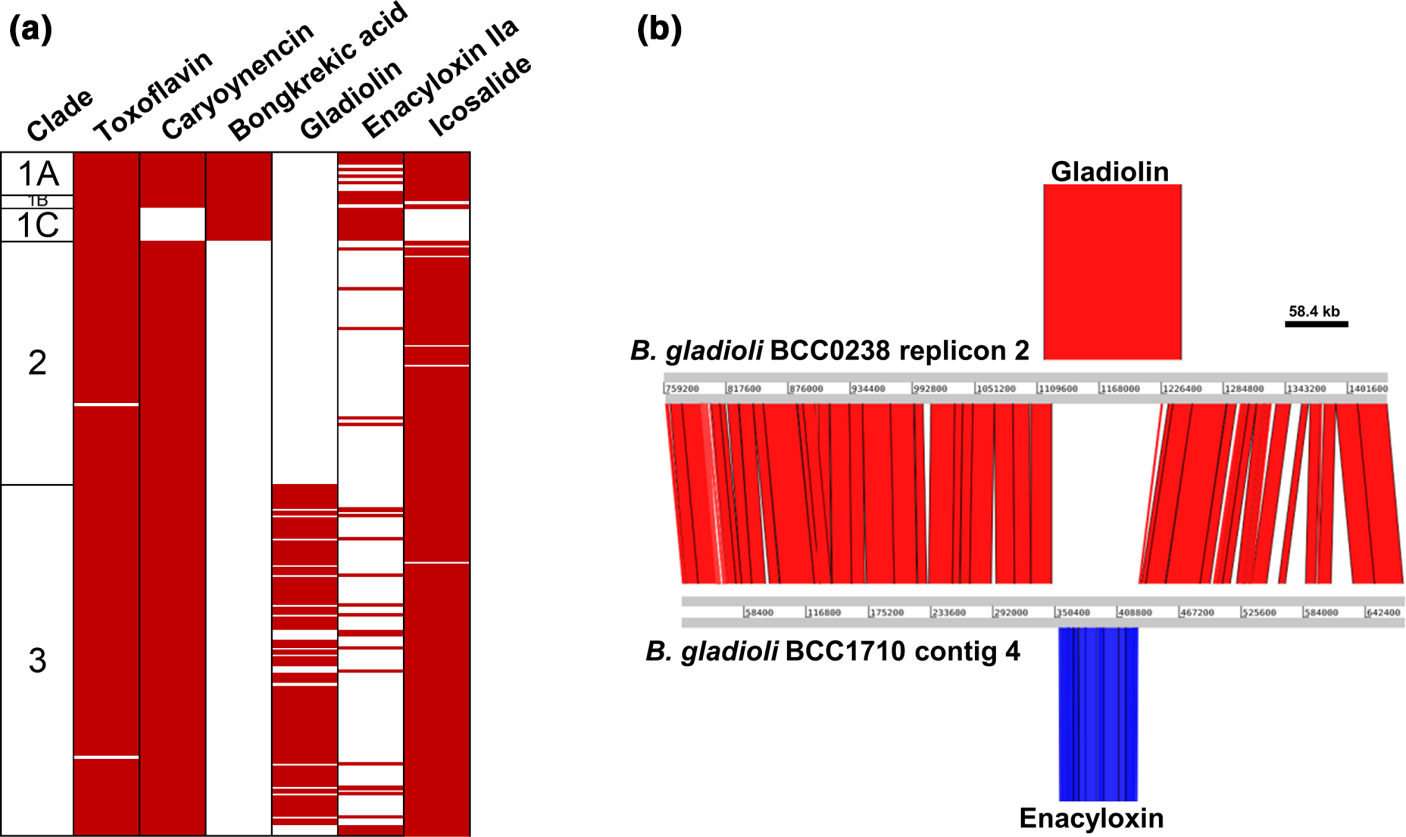
Distribution of known *B. gladioli* specialized metabolite BGCs and common genomic location for gladiolin and enacyloxin biosynthesis. (a) Sequence reads were mapped to known specialized metabolite BGCs to determine their presence or absence within the 206 the *B. gladioli* genomes. BGC presence indicated by red shading and columns from left to right show the *B. gladioli* clade, and BGCs for toxoflavin, caryoynencin, bongkrekic acid, gladiolin, enacyloxin IIa and icosalide. (b) A genomic comparison plot was constructed using the Artemis Comparison Tool for *B. gladioli* BCC0238 (clade 3) and BCC1710 (clade 1A). The common insertion point for either the gladiolin or enacyloxin BGCs in these strains is shown, together with extensive genomic synteny upstream and downstream of this specialized metabolite-encoding location (scale bar indicates 58.4 kb).

To characterize the genetic basis for the observed bioactivity and metabolite production profiles (Fig. 4), the distribution of specialized metabolite BGC was investigated within the *B. gladioli* genome collection. Using sequence read mapping to *B. gladioli* BGCs for known metabolites enabled toxoflavin, caryoynencin, bongrekic acid, enacyloxin, gladiolin and icosalide BGCs to be mapped. Across the phylogenomically defined *B. gladioli* clades, random distribution of BGCs was observed in some cases, whereas other BGCs were found to be clade-specific (Fig. 5a). The toxoflavin, caryoynencin and icosalide BGCs were widely distributed across *B. gladioli *, with the toxoflavin BGC being absent from only 2 of the 206 strains. The caryoynencin BGC was uniquely absent from all 10 clade 1C strains (correlating to a lack of detection of the metabolite; Fig. 4b). The icosalide BGC mirrored this clade 1C absence, but also showed random loss in six other strains from across *B. gladioli* (one clade 1B, four clade 2 and one clade 3 strain; Fig. 5a). The gladiolin and bongkrekic BGCs demonstrated evolutionary restrictions to specific clades as follows. A total of 83 of the 106 clade 3 strains (78%) contained the gladiolin BGC and it was absent from all other *B. gladioli* clades. All 27 strains within clades 1A, 1B and 1C contained the bongkrekic acid BGC, validating its presence as a marker that collectively designates them as group 1 strains (Fig. 1), despite their distinct nature as evolutionary clades (Fig. 2).

The enacyloxin BGC was randomly distributed across *B. gladioli* (Fig. 5a). Its presence in clade 1 strains was most conserved with 21 of 27 strains (77 %) and 100 % of clade 1C strains contained the BGC. Interestingly, no strain within the collection of 206 contained both the enacyloxin and gladiolin BGCs (Fig. 5a). Genomic interrogation of this inverse correlation led to the discovery that these large polyketide BGCs occupied the same genetic locus in *B. gladioli* (Fig. 5b). This conserved region of the genome is on the second genomic replicon of *B. gladioli* and contains either enacyloxin (43 strains), gladiolin (83 strains), or no specialized metabolite (80 strains) BGCs. Upstream and downstream of this polyketide BGC insertion point were blocks of conserved and syntenic genomic DNA. These surrounding regions of the *B. gladioli* second genomic replicon did not contain mobile DNA markers, indicating the BGC insertion point as a gene capture hotspot.

### *B. gladioli* bongkrekic acid biosynthesis: a new potential risk factor for CF lung infection

In total, 25 of the 27 strains in clades 1A, 1B and 1C were recovered from lung infections in people with CF (Table S1, Fig. 2), and all possess the bongkrekic acid BGC (Fig. 5a). To date this lethal toxin has only been associated with *B. gladioli *-related food poisoning [13, 14, 49], and has not been linked to disease in people with CF. Analysis of 12 toxin BGC-positive strains showed that 11 of them produced bongkrekic acid *in vitro*, but to varying extents (Fig. S4). Four of these *B. gladioli* strains (BCC1675, BCC1686, BCC1701 and BCC1710; Fig. S4) were subsequently grown in artificial CF sputum medium [27], and with the exception of strain BCC1701 (a low toxin producer; Fig. S4), toxin production was detected by high-resolution LC-MS. This observation prompted the development of a diagnostic PCR to enable rapid identification of *B. gladioli* isolates containing the bongkrekic acid BGC, as a potential clinical risk marker for CF. Application of this PCR (Fig. S5) to 122 *B. gladioli* CF isolates prior to their genome sequencing identified 13 positives. Subsequent genome sequencing demonstrated that all contained a complete bongkrekic acid BGC (Fig. 5a), validating the approach.

**Fig. 6.**
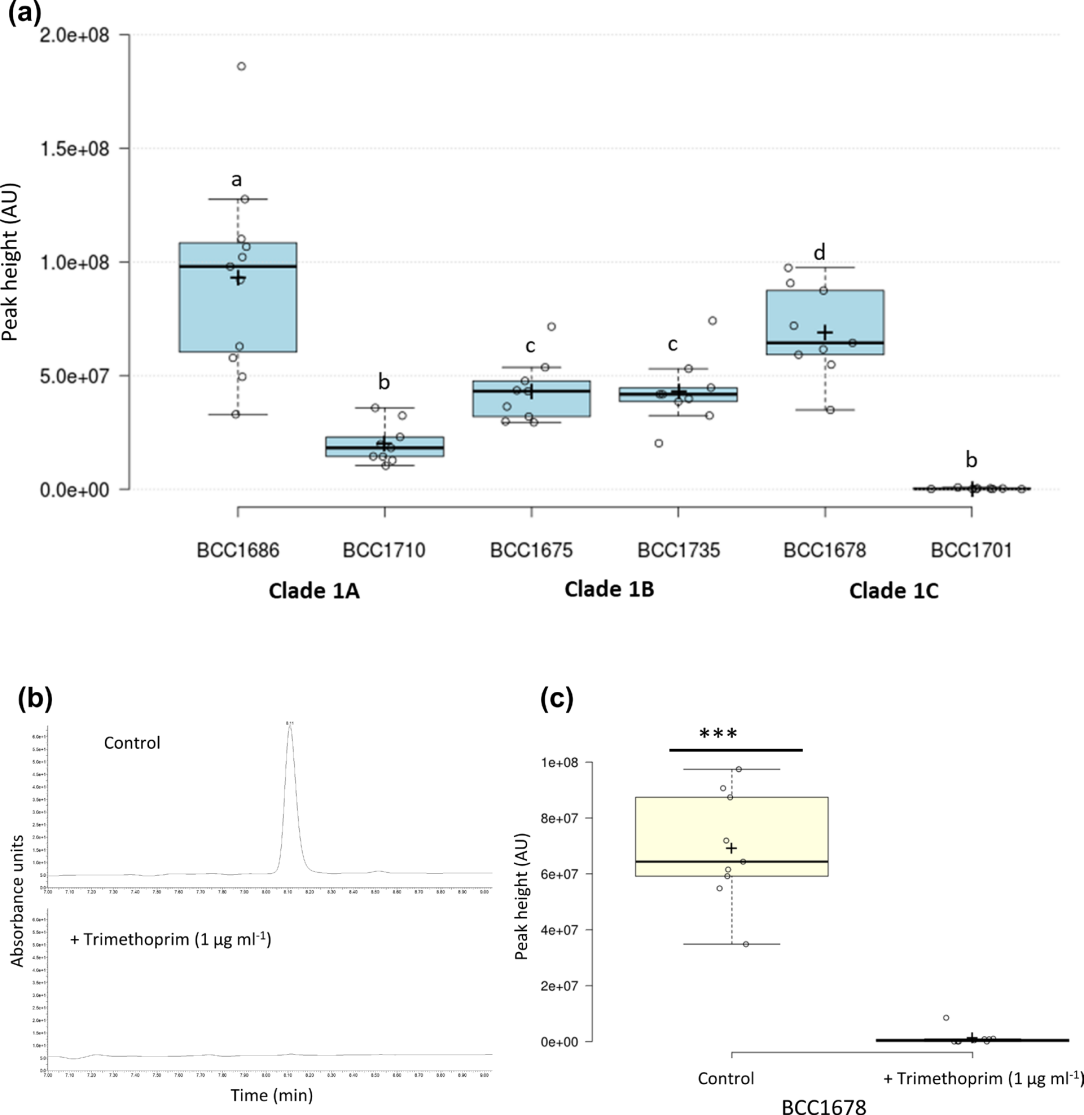
Sub-inhibitory concentrations of trimethoprim reduce bongkrekic acid production by *B. gladioli* clade 1 strains. (a) HPLC analysis of 6 *B. gladioli* strains (strain numbers are shown) belonging to clades 1A, 1B and 1C demonstrated variable levels of bongkrekic acid toxin production by HPLC analysis of their metabolite extracts (peak height is plotted). Differences between the mean values of bongkrekic acid were determined using the LSD test α=0.05. Means followed by the same letter are not significantly different. (b) The presence of sub-inhibitory concentrations of trimethoprim (1 µg ml^−1^) reduced the production of bongkrekic acid in strain BCC1678, as shown by the HPLC metabolite analysis comparing production levels against the control condition without antibiotic. (c) Quantitative comparison of bongkrekic acid production by strain BCC1678 in the presence and absence of trimethoprim (*n*=9) shows the significant (*P*<0.001) suppression of toxin biosynthesis caused by antibiotic exposure. Statistical significance was determined using a two-tailed *t*-test.

Since CF patients are administered multiple antibiotics to suppress lung infections, a recent report that low concentrations of antibiotics may induce specialized metabolite production in *Burkholderia* [50] further highlighted the potential risk of bongkrekic acid-producing *B. gladioli* strains to CF patients. Furthermore, the antibiotic trimethoprim, which is widely used for treatment of *Burkholderia* infections in CF, was shown to be an effective elicitor of specialized metabolite production in *B. thailandensis* [28], compounding the threat of strains containing the bongkrekic acid BGC. To examine whether trimethoprim induced expression of the bongkrekic acid BGC, six *B. gladioli* strains isolated from CF patients that produced bongkrekic acid at a range of titres (Fig. 6a) were investigated to determine whether sub-inhibitory levels of the antibiotic increased toxin biosynthesis. In contrast to *B. thailandensis* [28], no *B. gladioli* strains demonstrated induction of bongkrekic acid upon exposure to trimethoprim. Instead, toxin production was suppressed in five of the six strains analysed (* B. gladioli* BCC1678; Figs 6b, c and S6 show the data for all six strains tested).

## Discussion

In the last decade, the specialized metabolites produced by *Burkholderia* have been studied extensively and multiple compounds have been shown to be functional in different ecological settings [15]. *B. gladioli* shows a very wide range of beneficial [9, 20, 21] versus detrimental traits [7, 8, 13, 49, 51], several of which relate to specialized metabolite production. With multiple taxonomic reclassifications [7, 9], an unknown basis for pathovar status in plant disease [7, 8, 11], calls for a specific recognition of the lethal food-poisoning *cocovenenans* pathovar [13], an emerging presence in CF lung infection [2] and an expanding role as a source of specialized metabolites [15, 17–19, 21, 48], there is a clear need to better understand the population ecology of *B. gladioli *.

The population biology and pathogenicity traits of very few *Burkholderia* species have been described in depth using phylogenomics. The transmission dynamics of a *Burkholderia dolosa* CF outbreak was tracked in 14 patients over 16 years and identified mutations in the 112 isolates that showed parallel evolution towards increased antibiotic resistance and tolerance of low oxygen [52]. The cause of melioidosis, *Burkholderia pseudomallei *, has been subjected to arguably the most extensive genomic characterization because of its pathogenicity and due to its threat as a bioterrorism agent [53]. Genome sequencing of 469 *B. pseudomallei* isolates showed the species comprised 2 distinct populations, an ancestral Australian reservoir that anthropogenically transmitted and diverged within Asia, and spread further via the slave trade from Africa to South America [53]. Mapping the phylogenomics of *B. ambifaria* as a historically used biopesticide identified cepacin as a key antimicrobial mediating plant protection against pathogenic oomycetes [22]. However, the population biology of plant pathogenic *Burkholderia* has not been studied and our genomic analysis of *B. gladioli* is unique in uncovering whether plant pathovar status has an evolutionary basis. Only the *B. gladioli* pv. *allicola* associated with onion soft-rot plant disease were evolutionarily distinct as clade 2 strains (Fig. 2), but rotting capability was associated with all clades and historical pathovars (Figs 3 and S1). The specific genetic factors linked to the separation of pathovar *allicola* as clade 2 and its distinction from clade 3 plant disease strains remain to be determined.

In contrast to the broad conservation of plant disease traits across *B. gladioli *, bongkrekic acid-producing strains [13, 14, 49] associated with fatal human food poisoning were more closely related. They were designated group 1 by their ANI relatedness (Fig. 1) and the conserved presence of the bongkrekic acid BGC in these strains adds weight to the call for their differentiation as a toxin-producing *B. gladioli* subgroup [13]. With 13 % of the *B. gladioli* CF isolates examined containing the bongkrekic acid BGC, toxin production is a worrying potential risk factor for CF lung infections, especially as this can occur under lung infection-like growth conditions, such as artificial CF sputum. The clinical outcome of *Burkholderia* infection is frequently highly variable [54] and severe systemic disease has been associated with *B. gladioli* in CF [5]. With the ability to rapidly identify bongkrekic acid-positive *B. gladioli* isolates using a PCR diagnostic (Fig. S5), we are now in a strong position to understand whether the toxin plays a role in poor clinical outcome for infected CF patients. Also, since we have shown that trimethoprim acts to suppress toxin production (Fig. 6), rather than activate it [27, 28], a case can be made for therapy with the antibiotic to be maintained in bongkrekic acid-positive *B. gladioli* CF infections.

By combining genomics with analytical chemistry, we have also been able to map the repertoire of bioactive specialized metabolite BGCs across the *B. gladioli* species. This demonstrated that the bioactivity of *B. gladioli* frequently results from the production of multiple metabolites (Fig. 4). The widespread distribution and conservation of BGCs for toxoflavin [16], caryoynencin [48] and the icosalides [18, 19] suggest that they are ancestral to *B. gladioli* as a species (Fig. 5). The confinement of the gladiolin BGC to clade 3 strains also sheds light on the classification of the recently identified *B. gladioli* symbiont strain that protected the eggs of Lagriinae beetles from fungal attack [20]. The beetle symbiont, *B. gladioli* Lv-StA, contains the gladiolin BGC [21] and has been reported to produce lagriene [20], also known as iso-gladiolin, which results from rearrangement of gladiolin during isolation [21]. Since the gladiolin BGC is restricted to *B. gladioli* clade 3, the characterized insect symbiont [20] must be a member of this clade. Whether other evolutionary groups within *B. gladioli* also form these close associations with insects remains to be determined. It is also clear that all *B. gladioli* clades are geographically widely distributed from the analysis of US CF infection strains (Fig. S2). The ecological significance of herbivorous Lagriinae and other beetles in distributing the such bacterial symbionts across continental ranges will be fascinating to understand.

We were able to gain an insight into the ecological distribution of *B. gladioli* by indirectly sampling the opportunistic infections the bacterium causes in people with CF. Although our *B. gladioli* collection was mainly derived from people with CF living in the USA (126 isolates; Table S1), the 206 genomes examined were genetically diverse and comprised 133 unique MLST sequence types (Table S1). In the absence of patient-to-patient or common source transmission, the natural environment is the main source of *Burkholderia* lung infections in CF patients [2]. From the US CF patient data, all *B. gladioli* clades appear widely distributed across the North American continental range (Fig. S2), with the majority being represented by genetically unique strains (Table S1). Soil, the rhizosphere and terrestrial freshwater environments are common sources of *Burkholderia* [3]. Outside of the infection of people with CF [2], plant disease [7, 8, 11] or food-poisoning [13], little is known about other sources of *B. gladioli *. Recent findings of close associations with insects [20] and fungi [18, 19] point to multiple symbiotic roles played by *B. gladioli* in the natural environment. By defining a systematic framework of population biology and metabolite production, a basis from which to understand the diverse ecology of *B. gladioli* is now in place.

## Supplementary Data

Supplementary material 1

## References

[1] Depoorter E, Bull MJ, Peeters C, Coenye T, Vandamme P (2016). *Burkholderia*: an update on taxonomy and biotechnological potential as antibiotic producers. Appl Microbiol Biotechnol.

[2] Lipuma JJ (2010). The changing microbial epidemiology in cystic fibrosis. Clin Microbiol Rev.

[3] Suárez-Moreno ZR, Caballero-Mellado J, Coutinho BG, Mendonça-Previato L, James EK (2012). Common features of environmental and potentially beneficial plant-associated *Burkholderia*. Microb Ecol.

[4] Estrada-de Los Santos P, Palmer M, Chávez-Ramírez B, Beukes C, Steenkamp ET (2018). Whole genome analyses suggests that *Burkholderia* sensu lato contains two additional novel genera (*Mycetohabitans* gen. nov., and *Trinickia* gen. nov.): Implications for the evolution of diazotrophy and nodulation in the *Burkholderiaceae*. Genes.

[5] Jones AM, Stanbridge TN, Isalska BJ, Dodd ME, Webb AK (2001). *Burkholderia gladioli*: recurrent abscesses in a patient with cystic fibrosis. J Infect.

[6] Murray S, Charbeneau J, Marshall BC, LiPuma JJ (2008). Impact of burkholderia infection on lung transplantation in cystic fibrosis. Am J Respir Crit Care Med.

[7] Coenye T, Holmes B, Kersters K, Govan JR, Vandamme P (1999). Burkholderia cocovenenans (van Damme *et al.* 1960) Gillis *et al.* 1995 and *Burkholderia vandii* Urakami *et al.* 1994 are junior synonyms of *Burkholderia gladioli* (Severini 1913) Yabuuchi *et al.* 1993 and *Burkholderia plantarii* (Azegami *et al.* 1987) Urakami *et al.* 1994, respectively. Int J Syst Bacteriol.

[8] Hildebrand DC, Palleroni NJ, Doudoroff M (1973). Synonymy of *Pseudomonas gladioli* Severini 1913 and *Pseudomonas marginata* (McCulloch 1921) Stapp 1928. Int J Syst Bacteriol.

[9] Coenye T, Gillis M, Vandamme P (2000). Pseudomonas antimicrobica Attafuah and Bradbury 1990 is a junior synonym of Burkholderia gladioli (Severini 1913) Yabuuchi et al. 1993. Int J Syst Evol Microbiol.

[10] Gill WM, Tsuneda A (1997). The interaction of the soft rot bacterium *Pseudomonas gladioli* pv. *agaricicola* with Japanese cultivated mushrooms. Can J Microbiol.

[11] Wright PJ, Clark RG, Hale CN (1993). A storage soft rot of New Zealand onions caused by *Pseudomonas gladioli* Pv *alliicola*. New Zeal J Crop Hort.

[12] Nandakumar R, Shahjahan AKM, Yuan XL, Dickstein ER, Groth DE (2009). *Burkholderia glumae* and *B. gladioli* Cause Bacterial Panicle Blight in Rice in the Southern United States. Plant Dis.

[13] Jiao Z, Kawamura Y, Mishima N, Yang R, Li N (2003). Need to differentiate lethal toxin-producing strains of *Burkholderia gladioli*, which cause severe food poisoning: description of *B. gladioli* pathovar cocovenenans and an emended description of *B. gladioli*. Microbiol Immunol.

[14] Moebius N, Ross C, Scherlach K, Rohm B, Roth M (2012). Biosynthesis of the respiratory toxin bongkrekic acid in the pathogenic bacterium *Burkholderia gladioli*. Chem Biol.

[15] Kunakom S, Eustáquio AS (2019). *Burkholderia* as a source of natural products. J Nat Prod.

[16] Lee J, Park J, Kim S, Park I, Seo Y-S (2016). Differential regulation of toxoflavin production and its role in the enhanced virulence of *Burkholderia gladioli*. Mol Plant Pathol.

[17] Ross C, Opel V, Scherlach K, Hertweck C (2014). Biosynthesis of antifungal and antibacterial polyketides by *Burkholderia gladioli* in coculture with *Rhizopus microsporus*. Mycoses.

[18] Dose B, Niehs SP, Scherlach K, Flórez LV, Kaltenpoth M (2018). Unexpected bacterial origin of the antibiotic icosalide: two-tailed depsipeptide assembly in multifarious *Burkholderia* symbionts. ACS Chem Biol.

[19] Jenner M, Jian X, Dashti Y, Masschelein J, Hobson C (2019). An unusual *Burkholderia gladioli* double chain-initiating nonribosomal peptide synthetase assembles 'fungal' icosalide antibiotics. Chem Sci.

[20] Flórez LV, Scherlach K, Gaube P, Ross C, Sitte E (2017). Antibiotic-producing symbionts dynamically transition between plant pathogenicity and insect-defensive mutualism. Nat Commun.

[21] Song L, Jenner M, Masschelein J, Jones C, Bull MJ (2017). Discovery and biosynthesis of gladiolin: A *Burkholderia gladioli* Antibiotic with promising activity against *Mycobacterium tuberculosis*. J Am Chem Soc.

[22] Mullins AJ, Murray JAH, Bull MJ, Jenner M, Jones C (2019). Genome mining identifies cepacin as a plant-protective metabolite of the biopesticidal bacterium *Burkholderia ambifaria*. Nat Microbiol.

[23] Seo Y-S, Lim J, Choi B-S, Kim H, Goo E (2011). Complete genome sequence of *Burkholderia gladioli* BSR3. J Bacteriol.

[24] Johnson SL, Bishop-Lilly KA, Ladner JT, Daligault HE, Davenport KW (2015). Complete genome sequences for 59 *Burkholderia* isolates, both pathogenic and near neighbor. Genome Announc.

[25] Mahenthiralingam E, Song L, Sass A, White J, Wilmot C (2011). Enacyloxins are products of an unusual hybrid modular polyketide synthase encoded by a cryptic *Burkholderia ambifaria* Genomic Island. Chem Biol.

[26] Hareland WA, Crawford RL, Chapman PJ, Dagley S (1975). Metabolic function and properties of 4-hydroxyphenylacetic acid 1-hydroxylase from *Pseudomonas acidovorans*. J Bacteriol.

[27] Kirchner S, Fothergill JL, Wright EA, James CE, Mowat E (2012). Use of artificial sputum medium to test antibiotic efficacy against *Pseudomonas aeruginosa* in conditions more relevant to the cystic fibrosis lung. J Vis Exp.

[28] Okada BK, Wu Y, Mao D, Bushin LB, Seyedsayamdost MR (2016). Mapping the Trimethoprim-induced secondary metabolome of *Burkholderia thailandensis*. ACS Chem Biol.

[29] Connor TR, Loman NJ, Thompson S, Smith A, Southgate J (2016). CLIMB (the cloud infrastructure for microbial bioinformatics): an online resource for the medical microbiology community. Microb Genom.

[30] Magoč T, Salzberg SL (2011). FLASH: fast length adjustment of short reads to improve genome assemblies. Bioinformatics.

[31] Bankevich A, Nurk S, Antipov D, Gurevich AA, Dvorkin M (2012). SPAdes: a new genome assembly algorithm and its applications to single-cell sequencing. J Comput Biol.

[32] Walker BJ, Abeel T, Shea T, Priest M, Abouelliel A (2014). Pilon: an integrated tool for comprehensive microbial variant detection and genome assembly improvement. PLoS One.

[33] Seemann T (2014). Prokka: rapid prokaryotic genome annotation. Bioinformatics.

[34] Delcher AL, Bratke KA, Powers EC, Salzberg SL (2007). Identifying bacterial genes and endosymbiont DNA with glimmer. Bioinformatics.

[35] Galardini M, Biondi EG, Bazzicalupo M, Mengoni A (2011). CONTIGuator: a bacterial genomes finishing tool for structural insights on draft genomes. Source Code Biol Med.

[36] Pritchard L, Glover RH, Humphris S, Elphinstone JG, Toth IK (2016). Genomics and taxonomy in diagnostics for food security: soft-rotting enterobacterial plant pathogens. Analytical Methods.

[37] Page AJ, Cummins CA, Hunt M, Wong VK, Reuter S (2015). Roary: rapid large-scale prokaryote pan genome analysis. Bioinformatics.

[38] Price MN, Dehal PS, Arkin AP (2010). FastTree 2--approximately maximum-likelihood trees for large alignments. PLoS One.

[39] Jolley KA, Maiden MCJ (2010). BIGSdb: scalable analysis of bacterial genome variation at the population level. BMC Bioinformatics.

[40] Weber T, Blin K, Duddela S, Krug D, Kim HU (2015). antiSMASH 3.0-A comprehensive resource for the genome mining of biosynthetic gene clusters. Nucleic Acids Res.

[41] Blin K, Medema MH, Kottmann R, Lee SY, Weber T (2017). The antiSMASH database, a comprehensive database of microbial secondary metabolite biosynthetic gene clusters. Nucleic Acids Res.

[42] Roy Chowdhury P, Heinemann JA (2006). The general secretory pathway of *Burkholderia gladioli* pv. agaricicola BG164R is necessary for cavity disease in white button mushrooms. Appl Environ Microbiol.

[43] Jacobs JL, Fasi AC, Ramette A, Smith JJ, Hammerschmidt R (2008). Identification and onion pathogenicity of *Burkholderia cepacia* complex isolates from the onion rhizosphere and onion field soil. Appl Environ Microbiol.

[44] Webster G, Jones C, Mullins AJ, Mahenthiralingam E (2020). A rapid screening method for the detection of specialised metabolites from bacteria: induction and suppression of metabolites from Burkholderia species. J Microbiol Methods.

[45] Flannagan RS, Aubert D, Kooi C, Sokol PA, Valvano MA (2007). *Burkholderia cenocepacia* requires a periplasmic HtrA protease for growth under thermal and osmotic stress and for survival in vivo. Infect Immun.

[46] Mullins AJ, Jones C, Bull MJ, Webster G, Parkhill J (2020). Genomic assemblies of members of *Burkholderia* and related genera as a resource for natural product discovery. Microbiol Resour Announc.

[47] Goris J, Konstantinidis KT, Klappenbach JA, Coenye T, Vandamme P (2007). DNA-DNA hybridization values and their relationship to whole-genome sequence similarities. Int J Syst Evol Microbiol.

[48] Ross C, Scherlach K, Kloss F, Hertweck C (2014). The molecular basis of conjugated polyyne biosynthesis in phytopathogenic bacteria. Angew Chem Int Ed *Engl*.

[49] Gudo ES, Cook K, Kasper AM, Vergara A, Salomão C (2018). Description of a mass poisoning in a rural district in Mozambique: the first documented bongkrekic acid poisoning in Africa. Clin Infect Dis.

[50] Seyedsayamdost MR (2014). High-throughput platform for the discovery of elicitors of silent bacterial gene clusters. Proc Natl Acad Sci U S A.

[51] Quon BS, Reid JD, Wong P, Wilcox PG, Javer A (2011). *Burkholderia gladioli* - a predictor of poor outcome in cystic fibrosis patients who receive lung transplants? A case of locally invasive rhinosinusitis and persistent bacteremia in a 36-year-old lung transplant recipient with cystic fibrosis. Can Respir J.

[52] Lieberman TD, Michel J-B, Aingaran M, Potter-Bynoe G, Roux D (2011). Parallel bacterial evolution within multiple patients identifies candidate pathogenicity genes. Nat Genet.

[53] Chewapreecha C, Holden MTG, Vehkala M, Välimäki N, Yang Z (2017). Global and regional dissemination and evolution of *Burkholderia pseudomallei*. Nat Microbiol.

[54] Frangolias DD, Mahenthiralingam E, Rae S, Raboud JM, Davidson AGF (1999). *Burkholderia cepacia* in cystic fibrosis. Am J Respir Crit Care Med.

