# Supplementary material for: Kill and cure: genomic phylogeny and bioactivity of Burkholderia gladioli bacteria capable of pathogenic and beneficial lifestyles
Source: Microb Genom. 2021 Jan 18;7(1):mgen000515. doi: 10.1099/mgen.0.000515 (PMC8115902; doi:10.1099/mgen.0.000515)
Supplement: Supplementary material 1 [file mgen-7-515-s001.pdf]

Supplementary Information

Kill and cure: genomic phylogeny and bioactivity of *Burkholderia gladioli* bacteria capable of pathogenic and beneficial lifestyles

Running title: Population genomics and bioactivity of *B. gladioli*

Authors:
Cerith Jones[a,e], Gordon Webster[a], Alex J. Mullins[a], Matthew Jenner[b,i], Matthew J. Bull[a,f], Yousef Dashti[b,g], Theodore Spilker[c], Julian Parkhill[d,h], Thomas R. Connor[a], John J. LiPuma[c], Gregory L. Challis[b,i,j] and Eshwar Mahenthiralingam[a#]

Affiliations:
[a] Microbiomes, Microbes and Informatics Group, Organisms and Environment Division, School of Biosciences, Cardiff University, Cardiff, Wales, CF10 3AX, UK

[b] Department of Chemistry and Warwick Integrative Synthetic Biology Centre, University of Warwick, CV4 7AL, UK

[c] Department of Pediatrics, University of Michigan Medical School, Ann Arbor, Michigan, USA.

[d] Wellcome Trust Sanger Institute, Wellcome Trust Genome Campus, Hinxton, Cambridge, CB10 1SA UK.

[e] Current address: School of Applied Sciences, Faculty of Computing, Engineering and Science, University of South Wales, Pontypridd, CF37 4BD, UK

[f] Current address: Pathogen Genomics Unit, Public Health Wales Microbiology Cardiff, University Hospital of Wales, Cardiff, CF14 4XW

[g] Current address: The Centre for Bacterial Cell Biology, Biosciences Institute, Medical School, Newcastle University, Newcastle upon Tyne, NE2 4AX, UK

[h] Current address: Department of Veterinary Medicine, Madingley Road, Cambridge, CB3 0ES

[i] Warwick Integrative Synthetic Biology Centre, University of Warwick, Coventry CV4 7AL, UK

[j] Department of Biochemistry and Molecular Biology, Biomedicine Discovery Institute, Monash University, Clayton, VIC 3800, Australia

[#]**Corresponding Author:** Prof. Eshwar Mahenthiralingam, Microbiomes, Microbes and Informatics Group, Organisms and Environment Division, School of Biosciences, Cardiff University, Sir Martin Evans Building, Room 2.06, Museum Avenue, Cardiff CF10 3AX, United Kingdom; Tel. +44 (0)29 20875875; Fax. +44 (0)29 20874305;
Email: MahenthiralingamE@cardiff.ac.uk (ORCID: 0000-0001-9014-3790)

Co-corresponding Author: Cerith Jones, School of Applied Sciences, Faculty of Computing, Engineering and Science, University of South Wales, Pontypridd, CF37 4BD, United Kingdom, Tel. +44 (0)1443 654711
Email: cerith.jones@southwales.ac.uk (ORCID 0000-0001-6275-023)

Key words: *Burkholderia, B. gladioli*, antibiotic production, plant pathogenesis, cystic fibrosis infection, phylogenomics

CONTENTS:

SUPPLEMENTARY TABLES

Table S1. Collection of *B. gladioli* isolates assembled and analysed in this study

Table S2. Primers and *B. gladioli* isolates used for generation of biosynthetic gene cluster insertional mutants

Table S3. Pan genome analysis metrics of *B. gladioli* and each core gene defined phylogenomic lineages

SUPPLEMENTARY FIGURES

Figure S1. *B. gladioli* from all evolutionary clades are capable of onion rot

Figure S2. CF infection associated with each *B. gladioli* clade is widely distributed across the United States of America

Figure S3. UHPLC-ESI-Q-TOF-MS analysis of metabolites produced by *B. gladioli* isolates

Figure S4. *B. gladioli* clade 1 strains have a variable ability to produce bongkrekic acid

Figure S5. Detection of *B. gladioli* clade 1 strains by bongkrekic acid *bonA* gene PCR

Figure S6. Subinhibitory trimethoprim reduces bongrekic acid production

Table S1: Collection of *B. gladioli* isolates assembled and analysed in this study

			Illumina Sequencing					Analysis Performed						
**Strain Name**	**Phylogenomic Clade**	**Strain Details** (CF = CF; ENV = environmental, ENVI = environmental from industry)	**Accession (PacBio)**	**Genome Size**	**Number of Contigs**	**%GC**	**N50**	**MLST sequence type**	**Core Genome Analysis**	**Mushroom Rot**	**Bioactivity Screening**	**HPLC Analysis**	**BGC Mapping**	**Bongkrekic acid analysis**
BCC1650	1A	ENV; *B. gladioli* pv *cocovenenans*, LMG 11626, Indonesia	ERS784828	8091943	35	68.24	708245	957				BCC1650		
BCC1661	1A	CF Isolate, USA	ERS784861	8327590	45	68.06	632785	959						
BCC1665	1A	CF Isolate, USA	ERS784925	8328710	45	68.06	601921	963						
BCC1675	1B	CF Isolate, USA	ERS785062	8080953	47	68.09	936495	970		BCC1675	BCC1675	BCC1675		BCC1675
BCC1678	1C	CF Isolate, USA	ERS784880	7819531	96	68.04	235084	973		BCC1678	BCC1678	BCC1678		BCC1678
BCC1686	1A	CF Isolate, USA	ERS784816	8334290	38	68.06	728975	963						BCC1686
BCC1689	1A	CF Isolate, USA	ERS784865	8252657	47	67.94	353209	993						
BCC1692	1A	CF Isolate, USA	ERS784914	8387798	41	67.98	810454	983						
BCC1697	1A	CF Isolate, USA	ERS784984	8085377	49	68.02	652756	989						
BCC1701	1C	CF Isolate, USA	ERS784867	8145614	94	67.98	280973	993				BCC1701		BCC1701
BCC1710	1A	CF Isolate, USA	ERS785039 (GCA_900608535)	8374934	44	68.02	615977	999		BCC1710	BCC1710	BCC1710		BCC1710
BCC1735	1B	CF Isolate, USA	ERS785008	7922226	64	67.98	388060	1019			BCC1735	BCC1735		BCC1735
BCC1766	1A	CF Isolate, USA	ERS1371589	8275704	144	67.89	275070	Novel						
BCC1780	1B	CF Isolate, USA	ERS1328777	8428886	208	67.76	220441	Novel						
BCC1781	1C	CF Isolate, USA	ERS1328887	7974402	195	68.16	208905	Novel						
BCC1812	1A	CF Isolate, USA	ERS1328895	8211259	162	67.94	301688	Novel						
BCC1819	1A	CF Isolate, USA	ERS1328796	8410357	115	67.9	331109	Novel						
BCC1821	1B	CF Isolate, USA	ERS1328797	8122457	126	68.08	263017	Novel						
BCC1829	1A	CF Isolate, USA	ERS1371601	8600125	112	67.8	317992	Novel						
BCC1837	1C	CF Isolate, USA	ERS1371606	8128598	159	67.99	206652	Novel						
BCC1843	1C	CF Isolate, USA	ERS1328808	7992663	209	68.1	177545	Novel						
BCC1844	1C	CF Isolate, USA	ERS1328903	8186135	206	67.98	219436	Novel						
BCC1861	1C	CF Isolate, USA	ERS1371619	8609383	249	67.75	216674	Novel						
BCC1864	1C	CF Isolate, USA	ERS1328908	8490658	220	67.9	212901	Novel						

BCC1870	1C	CF Isolate, USA	ERS1371625	8341408	182	67.95	186380	Novel						
BCC1871	1C	CF Isolate, USA	ERS1371626	8043875	155	68.11	284538	Novel			BCC1871	BCC1871		
BCC1880	1A	ENV; *B. gladioli* pv *cocovenenans*, LMG 18113, China	ERS1371628	8222883	77	68.04	621809	Novel						
BCC0252	2	CF Isolate, Australia	ERS784955	8212491	63	68.09	289654	947						
BCC1619	2	CF Isolate, USA	ERS784817	7880197	36	68.13	868935	554						
BCC1620	2	CF Isolate, USA	ERS784832	8184546	61	68.09	416357	951						
BCC1621	2	CF Isolate, USA	ERS784849 (GCA_900608525)	8467292	76	67.95	434143	903						
BCC1624	2	CF Isolate, USA	ERS784895	7876116	48	68.14	430243	554						
BCC1645	2	ENV; *B. gladioli* pv. *alliicola*, LMG 6954, Australia	ERS784911	8603880	78	67.87	383325	903				BCC1645		
BCC1646	2	ENV; *B. gladioli* pv. *alliicola*, LMG 6878, India	ERS784928	8379242	87	67.96	286552	954						
BCC1648	2	ENV; *B. gladioli* pv. *alliicola*, LMG 2121, USA	ERS784957	8607718	85	67.87	310631	903						
BCC1662	2	CF Isolate, USA	ERS784878	8507695	67	68.05	410006	947						
BCC1663	2	CF Isolate, USA	ERS784894	8510108	64	68.05	410015	947						
BCC1664	2	CF Isolate, USA	ERS784910	8498792	73	68.06	378407	947						
BCC1666	2	CF Isolate, USA	ERS784942	8506933	67	68.05	408038	947						
BCC1667	2	CF Isolate, UK	ERS784960	8480007	78	67.84	427447	965						
BCC1670	2	CF Isolate, UK	ERS784814	8282986	75	68.03	309426	903						
BCC1671	2	CF Isolate, UK	ERS784830	8347055	76	68.09	369886	968						
BCC1672	2	CF Isolate, UK	ERS784846	8272557	75	68.03	287015	903						
BCC1674	2	CF Isolate, UK	ERS784994	8431622	84	67.87	356821	965						
BCC1676	2	CF Isolate, USA	ERS784863	7987029	52	68.26	467101	971						
BCC1679	2	CF Isolate, USA	ERS784896	8113442	62	68.09	272665	971						
BCC1680	2	CF Isolate, USA	ERS784912	7965535	52	68	411881	554						
BCC1681	2	CF Isolate, USA	ERS784927	7321015	56	68.09	382283	975				BCC1681		
BCC1682	2	CF Isolate, USA	ERS784944	7942355	57	68.03	428648	947						
BCC1684	2	CF Isolate, USA	ERS784975	8383929	56	67.96	576064	971						
BCC1685	2	CF Isolate, USA	ERS784983	7943459	66	68.03	314381	947						
BCC1690	2	CF Isolate, USA	ERS784881	8470418	75	67.94	433041	903						
BCC1693	2	CF Isolate, USA	ERS784929	8210869	47	68.1	668608	554						
BCC1695	2	CF Isolate, USA	ERS784964	7905325	53	68.06	538303	971						

BCC1699	2	CF Isolate, USA	ERS784834	8345011	78	67.87	354820	991						
BCC1705	2	CF Isolate, USA	ERS784916	8145336	49	68.17	551683	995						
BCC1708	2	CF Isolate, USA	ERS784966	8331226	49	68.03	945197	554						
BCC1711	2	CF Isolate, USA	ERS785006	8344918	67	68.06	335836	903						
BCC1712	2	CF Isolate, USA	ERS785025	8201991	62	68.08	388388	971						
BCC1715	2	CF Isolate, USA	ERS784977	7963860	43	68.14	954999	1003						
BCC1716	2	CF Isolate, USA	ERS785052	8495662	77	67.87	385075	554						
BCC1717	2	CF Isolate, USA	ERS785014	8418479	69	67.93	430020	1004						
BCC1719	2	CF Isolate, USA	ERS785042	7988187	68	68.17	429154	903						
BCC1731	2	CF Isolate, USA	ERS784997	8485637	96	67.97	339104	1016		BCC1731				
BCC1756	2	CF Isolate, USA	ERS1328753	7729600	167	68.11	264066	903						
BCC1759	2	CF Isolate, USA	ERS1333276	8389900	206	67.93	202663	Novel						
BCC1761	2	CF Isolate, USA	ERS1371587	8105698	114	68.15	227067	Novel						
BCC1762	2	CF Isolate, USA	ERS1328756	8362320	214	67.86	232045	Novel						
BCC1764	2	CF Isolate, USA	ERS1371588	7946735	117	68.19	273951	Novel						
BCC1768	2	CF Isolate, USA	ERS1371591	8251454	139	68.11	226248	Novel						
BCC1769	2	CF Isolate, USA	ERS1328884	8343545	225	68.02	248528	554						
BCC1770	2	CF Isolate, USA	ERS1328772	8405384	148	67.98	261993	554			BCC1770	BCC1770		
BCC1771	2	CF Isolate, USA	ERS1328943	8398051	128	67.99	303762	554						
BCC1773	2	CF Isolate, USA	ERS1328885	8072772	131	68.21	264067	903						
BCC1774	2	CF Isolate, USA	ERS1371592	7885091	119	68.1	264045	903						
BCC1779	2	CF Isolate, USA	ERS1328944	8227602	296	68.02	190616	903						
BCC1782	2	CF Isolate, USA	ERS1328778	7984599	133	68.24	264129	971						
BCC1783	2	CF Isolate, USA	ERS1336060	8376932	184	67.96	224024	965						
BCC1785	2	CF Isolate, USA	ERS1328779	7979574	139	68.26	243147	971						
BCC1790	2	CF Isolate, USA	ERS1333278	8396898	197	67.9	249452	903						
BCC1792	2	CF Isolate, USA	ERS1328890	8115078	129	68.17	276382	1508						
BCC1795	2	CF Isolate, USA	ERS1328784	7813137	123	68.17	275050	Novel						
BCC1798	2	CF Isolate, USA	ERS1371596	8241429	151	68.07	230356	971						
BCC1803	2	CF Isolate, USA	ERS1371597	7705345	124	68.12	272468	554						
BCC1804	2	CF Isolate, USA	ERS1371598	8439232	162	68	250984	971						
BCC1805	2	CF Isolate, USA	ERS1328789	7850130	140	67.94	270833	554						

BCC1814	2	CF Isolate, USA	ERS1336062	8201270	156	68.14	206859	975						
BCC1815	2	CF Isolate, USA	ERS1328794	8940443	186	67.39	262346	954						
BCC1820	2	CF Isolate, USA	ERS1328897	8038288	131	68.15	242112	Novel						
BCC1825	2	CF Isolate, USA	ERS1328799	8200736	119	67.96	211609	971						
BCC1828	2	CF Isolate, Canada	ERS1328899	8234597	153	68.04	268035	971						
BCC1831	2	CF Isolate, Canada	ERS1371603	8416509	153	68	189469	947						
BCC1832	2	CF Isolate, USA	ERS1371604	8156592	135	68.08	208866	971						
BCC1834	2	CF Isolate, USA	ERS1328950	7897744	118	68.06	286322	991						
BCC1835	2	CF Isolate, USA	ERS1371605	8392649	159	67.96	264845	554						
BCC1838	2	CF Isolate, USA	ERS1336064	8426173	204	67.91	179624	947						
BCC1848	2	CF Isolate, USA	ERS1328904	8130201	112	68.04	301164	Novel			BCC1848	BCC1848		
BCC1855	2	CF Isolate, USA	ERS1371614	8309427	177	68.01	186469	Novel						
BCC1865	2	CF Isolate, USA	ERS1328819	8217507	131	68.15	233312	971						
BCC1867	2	CF Isolate, USA	ERS1371623	8391961	158	67.96	178703	Novel						
BCC0238	3	CF Isolate, Canada	ERS784907 (GCA_900631635.1)	8541552	79	68	479899	946		BCC0238	BCC0238	BCC0238		
BCC0507	3	CF Isolate, Italy	ERS784970	8350939	61	68.13	335266	948						
BCC0771	3	*B. gladioli* pv. *gladioli*, LMG 2216^T (ATCC 10248), USA	ERS784806	8791975	51	67.72	967784	557						
BCC1317	3	ENVI , UK	ERS785017	8761762	63	67.93	552562	949						
BCC1622	3	CF Isolate, USA	ERS784864 (GCA_900608515)	8410623	51	68.05	615150	952						
BCC1623	3	CF Isolate, USA	ERS784879	8430801	78	67.87	365793	953						
BCC1647	3	*B. gladioli* pv. *gladioli*, LMG 6882, USA	ERS784943	8875626	51	67.7	561180	955						
BCC1649	3	ENV; *B. gladioli* pv. *gladioli*, LMG 6880t4, Zimbabwe	ERS784812	8241275	51	68.3	554529	956						
BCC1651	3	CF Isolate, LMG 18157	ERS784844	7931757	44	68.29	595470	958						
BCC1668	3	CF Isolate, UK	ERS784974	8456480	50	67.97	603154	966						
BCC1669	3	CF Isolate, UK	ERS784982	8173618	52	68.14	485051	967						
BCC1677	3	CF Isolate, USA	ERS785071	8109976	41	68.25	632433	972						
BCC1683	3	CF Isolate, USA	ERS784962	8306965	44	68.17	886986	977						
BCC1687	3	CF Isolate, USA	ERS784833	8185231	63	68.21	336464	981						
BCC1688	3	CF Isolate, USA	ERS784848	8455786	75	68.14	319266	982						
BCC1691	3	CF Isolate, USA	ERS784898	8419445	58	68.04	1964774	952						

BCC1694	3	CF Isolate, USA	ERS784946	8468446	45	67.9	688705	986						
BCC1696	3	CF Isolate, USA	ERS784976	8266956	75	68.19	429819	988						
BCC1698	3	CF Isolate, USA	ERS784818	8489077	68	68.1	434629	990						
BCC1700	3	CF Isolate, USA	ERS784851	8488515	43	68.08	1416119	992						
BCC1703	3	CF Isolate, USA	ERS785056	8286893	43	68.22	599795	955						
BCC1707	3	CF Isolate, USA	ERS784948	8009488	42	68.22	534508	997						
BCC1709	3	CF Isolate, USA	ERS785035	8457160	80	68.12	414686	998						
BCC1713	3	CF Isolate, USA	ERS785074	8456910	192	68.05	147146	1001				BCC1713		
BCC1714	3	CF Isolate, USA	ERS785080	8498126	70	68.08	361363	1002						
BCC1720	3	CF Isolate, USA	ERS784985	7902991	43	68.29	770588	958						
BCC1721	3	CF Isolate, USA	ERS784820	8216814	30	68.23	1289329	1006						
BCC1722	3	CF Isolate, USA	ERS784836	7994327	63	68.18	346974	1007						
BCC1723	3	CF Isolate, USA	ERS784853	8184213	44	68.25	1570053	1008						
BCC1724	3	CF Isolate, USA	ERS785060	8510702	64	68.03	505438	1006						
BCC1725	3	CF Isolate, USA	ERS784869	8559474	62	68.03	521790	1010						
BCC1726	3	CF Isolate, USA	ERS784885	8129584	39	68.29	848284	1011						
BCC1727	3	CF Isolate, USA	ERS785068	8153985	43	68.29	921754	1012						
BCC1728	3	CF Isolate, USA	ERS785003	8123767	50	68.29	391569	1013						
BCC1729	3	CF Isolate, USA	ERS784902	8365875	53	67.89	662719	1014						
BCC1730	3	CF Isolate, Canada	ERS784917	8528922	44	68.12	793173	1015						
BCC1732	3	CF Isolate, USA	ERS784933	8810912	64	67.67	606420	1017						
BCC1733	3	CF Isolate, USA	ERS784951	8462420	53	68.03	519208	1018						
BCC1734	3	CF Isolate, USA	ERS785079	8187286	59	68.24	757647	901						
BCC1754	3	CF Isolate, USA	ERS1371583	8372702	104	68.2	416499	Novel						
BCC1755	3	CF Isolate, USA	ERS1328941	8248941	166	68.2	324811	Novel						
BCC1757	3	CF Isolate, USA	ERS1371584	8106595	108	68.25	281288	Novel						
BCC1758	3	CF Isolate, USA	ERS1371585	8411592	135	68.16	347044	Novel						
BCC1760	3	CF Isolate, USA	ERS1371586	8142112	101	68.21	351231	972						
BCC1763	3	CF Isolate, USA	ERS1328942	8711025	150	68.08	409905	Novel						
BCC1765	3	CF Isolate, USA	ERS1328883	8049558	131	68.1	267531	Novel						
BCC1767	3	CF Isolate, USA	ERS1371590	8144838	89	68.28	365649	Novel						
BCC1772	3	CF Isolate, USA	ERS1328773	8423448	169	68.03	340707	Novel						

BCC1775	3	CF Isolate, USA	ERS1333277	8368392	155	68.13	332589	992						
BCC1777	3	CF Isolate, USA	ERS1328886	7915650	135	68.09	275756	Novel						
BCC1778	3	CF Isolate, USA	ERS1328776	8287507	135	68.04	284651	Novel						
BCC1784	3	CF Isolate, USA	ERS1328888	8106978	167	68.17	273802	Novel						
BCC1786	3	CF Isolate, USA	ERS1328945	8316388	145	68.18	347752	Novel						
BCC1787	3	CF Isolate, USA	ERS1371593	8239682	112	68.27	371296	Novel						
BCC1788	3	CF Isolate, USA	ERS1371594	8626684	376	67.84	291652	Novel						
BCC1789	3	CF Isolate, USA	ERS1328781	8320110	145	68.05	274262	901						
BCC1791	3	CF Isolate, USA	ERS1328782	8138900	125	68.14	334966	Novel						
BCC1793	3	CF Isolate, USA	ERS1328783	8137484	108	68.24	280591	Novel						
BCC1794	3	CF Isolate, USA	ERS1328946	8325891	167	68.23	281967	Novel						
BCC1796	3	CF Isolate, USA	ERS1328891	8374863	109	68.08	351151	902						
BCC1799	3	CF Isolate, USA	ERS1328786	8102290	84	68.22	343906	1015						
BCC1800	3	CF Isolate, USA	ERS1328892	7908681	135	68.29	257500	Novel				BCC1800		
BCC1801	3	CF Isolate, USA	ERS1328787	7951550	97	68.24	316850	958						
BCC1802	3	CF Isolate, USA	ERS1328947	8450656	130	68.11	270804	Novel						
BCC1806	3	CF Isolate, USA	ERS1333279	8476169	174	68.05	273269	992						
BCC1807	3	CF Isolate, USA	ERS1371599	8465961	109	68	304296	Novel						
BCC1808	3	CF Isolate, USA	ERS1371600	7962624	122	68.22	293939	901						
BCC1809	3	CF Isolate, USA	ERS1328791	8633417	162	67.87	235923	Novel						
BCC1810	3	CF Isolate, USA	ERS1328948	8276123	94	68.22	310714	955						
BCC1811	3	CF Isolate, USA	ERS1328792	8596479	208	67.93	305514	Novel						
BCC1813	3	CF Isolate, USA	ERS1328793	8559601	130	68.01	293202	Novel						
BCC1816	3	CF Isolate, Canada	ERS1328896	8179064	97	68.12	326051	Novel						
BCC1817	3	CF Isolate, USA	ERS1328795	8110913	127	68.21	242936	Novel						
BCC1818	3	CF Isolate, USA	ERS1333280	8154489	96	68.23	345879	1010						
BCC1822	3	CF Isolate, USA	ERS1336063	8078754	104	68.18	402601	Novel						
BCC1823	3	CF Isolate, USA	ERS1328798	8207755	107	68.31	336871	Novel						
BCC1826	3	CF Isolate, USA	ERS1328949	8374984	115	68.13	338267	Novel						
BCC1827	3	CF Isolate, USA	ERS1328800	8704353	169	67.88	269894	Novel						
BCC1830	3	CF Isolate, USA	ERS1371602	8388969	115	68.05	419764	Novel						
BCC1833	3	CF Isolate, USA	ERS1328803	8478257	126	68.02	390244	Novel						

BCC1836	3	CF Isolate, USA	ERS1328901	8169254	114	68.3	407323	Novel						
BCC1841	3	CF Isolate, USA	ERS1328807	8713715	196	67.94	274939	955						
BCC1842	3	CF Isolate, USA	ERS1328951	8157581	116	68.27	344808	Novel						
BCC1845	3	CF Isolate, USA	ERS1328809	8127806	86	68.24	362515	902						
BCC1846	3	CF Isolate, USA	ERS1371607	8405902	113	68	320953	Novel						
BCC1847	3	CF Isolate, USA	ERS1371608	8398980	143	68.17	287220	Novel						
BCC1849	3	CF Isolate, USA	ERS1371609	8211748	118	68.29	368142	Novel						
BCC1850	3	CF Isolate, USA	ERS1371610	8519442	175	68.02	275270	977						
BCC1851	3	CF Isolate, USA	ERS1371611	8365451	131	68.2	304291	Novel						
BCC1852	3	CF Isolate, USA	ERS1371612	8412916	113	68.05	333375	902						
BCC1853	3	CF Isolate, USA	ERS1328813	8212875	114	68.2	326166	Novel						
BCC1854	3	CF Isolate, USA	ERS1371613	8895140	180	67.81	274690	Novel						
BCC1856	3	CF Isolate, USA	ERS1328906	8038261	129	68.29	286277	Novel						
BCC1857	3	CF Isolate, USA	ERS1371615	8229080	111	68.25	280379	Novel						
BCC1858	3	CF Isolate, USA	ERS1371616	8186921	115	68.22	307394	Novel						
BCC1859	3	CF Isolate, USA	ERS1371617	8251063	116	68.2	334836	1010						
BCC1860	3	CF Isolate, USA	ERS1371618	8617021	136	68	347760	952			BCC1860	BCC1860		
BCC1862	3	CF Isolate, USA	ERS1371620	8378040	108	68.14	294407	Novel						
BCC1863	3	CF Isolate, USA	ERS1371621	8264387	121	68.23	294023	Novel						
BCC1866	3	CF Isolate, USA	ERS1371622	8450941	143	67.99	287257	1002						
BCC1868	3	CF Isolate, USA	ERS1371624	7929417	92	68.19	393583	Novel						
BCC1869	3	CF Isolate, USA	ERS1328821	8594173	159	67.92	311968	902						
BCC1872	3	CF Isolate, USA	ERS1371627	8415473	162	68.15	286407	Novel						
BCC1881	3	ENV; *B. gladioli* pv. *agaricicola*, NCPPB 3580, UK	ERS1371629	8378078	125	68.2	350910	Novel						
BCC1882	3	ENV; *B. gladioli* pv. *agaricicola*, NCPPB 3632, UK	ERS1371630	8374159	130	68.2	350910	Novel						
BCC1883	3	ENV; *B. gladioli* pv. *agaricicola*, NCPPB 3852, New Zeland	ERS1371631	8116617	99	68.31	603194	Novel		BCC1883	BCC1883	BCC1883		

Table S2. Primers and *B. gladioli* isolates used for generation of biosynthetic gene cluster insertional mutants

Target Cluster	Target gene	Strain	Forward Primer (5'-3')[a]	Reverse Primer (5'-3')[a]	Reference
Gladiolin	*gbnD1*	BCC0238	ATTTCTAGAGTTGCTGATC GACGCCTATC	TATGAATTCCGCCATAACC GAACGAACTC	Song *et al.* 2017
Toxoflavin	*toxA*	BCC0238	GACATCTAGATTGGTGATG TTTCCGGCAAG	GGCGGGTACCGAACACGT CCCAGAAACCAG	This work
Bongkrekic acid	*bonA*	BCC1710	ATTTCTAGAAGTATCCGCA TTTTCGTCGC	TATGAATTCGATCGATCAG TTGCGCTTCC	This work
Caryoynencin	*cayA*	BCC1697	GCTCTAGACATGTCGTGAT AAGGCGGC	GCGGAATTCACCGTGAGC TATGCCGAG	This work

Footnote: [a] The underlined primer sequence indicates the restriction enzyme site overlap used for cloning the PCR products into pGpΩTp

Table S3: Pan genome analysis metrics of *B. gladioli* and each core gene defined phylogenomic lineages

**Genome content** (definition)	***B. gladioli* phylogenomic lineages** (number of genomes)			**Sub-analysis of Group 1 phylogenomic lineages** (number of genomes)		
	**Group 1** (n=27)	**Group 2** (n=73)	**Group 3** (n=106)	**Group 1A** (n=13)	**Group 1B** (n=4)	**Group 1C** (n=10)
**Core** (99% <= strains <= 100%)	4847	4682	5109	5780	6011	5359
**Soft Core** (95% <= strains < 99%)	320	898	458	0	0	0
**Shell** (15% <= strains < 95%)	3096	2340	2489	2463	2633	2565
**Cloud genes** (0% <= strains < 15%)	7596	10487	14840	2237	0	3471
**Total**	15859	18407	22896	10480	8644	11395

SUPPLEMENTARY FIGURES

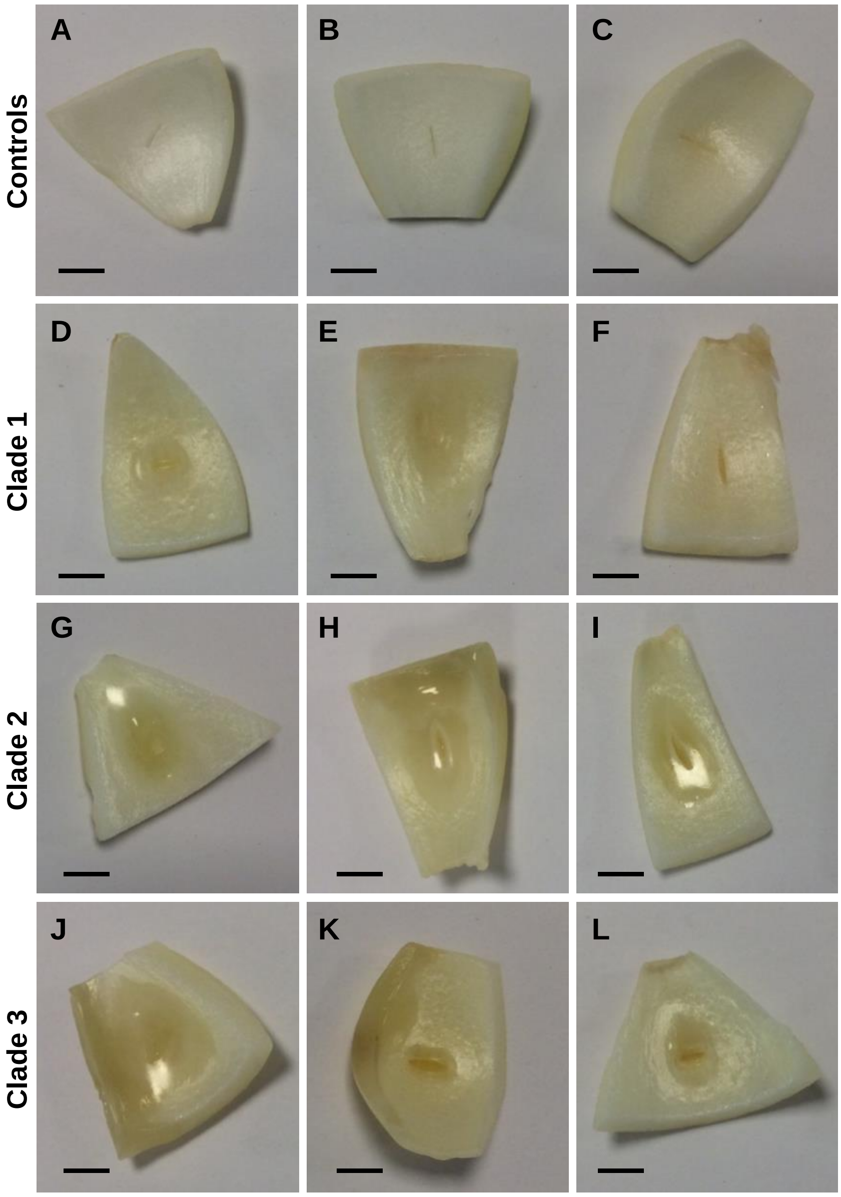

Figure S1. *B. gladioli* from all evolutionary clades are capable of onion rot. The ability of *B. gladioli* strains to degrade onion tissue was tested by inoculating injured onion tissue with standardized bacterial cultures. Inoculated mushrooms were incubated at 30°C for 48 hours with the following shown in each panel (each row are either controls or *B. gladioli* clades as indicated on the left): (A) No treatment control, (B) TSB only, (C) *E. coli* NCTC 12241, (D) *B. gladioli* Clade 1A strain BCC1710, (E) Clade 1B strain BCC1675, (F) Clade 1C strain BCC1678, (G) *B. gladioli* Clade 2 strain BCC1731, (H) *B. gladioli pv. allicola* reference Clade 2 strain BCC1645, (I) *B. gladioli pv. allicola* reference Clade 2 strain BCC1646, (J) *B. gladioli* Clade 3 strain BCC0238, (K) *B. gladioli pv. agaricola* reference Clade 3 strain BCC1883 (NCPPB 3852), (L) *B. gladioli pv. gladioli* reference Clade 3 strain BCC771 (LMG 2216^T). Pitting and tissue degradation was apparent in all *B. gladioli* inoculated mushrooms; a scale bar (1 cm) is shown in each panel to enable comparison.

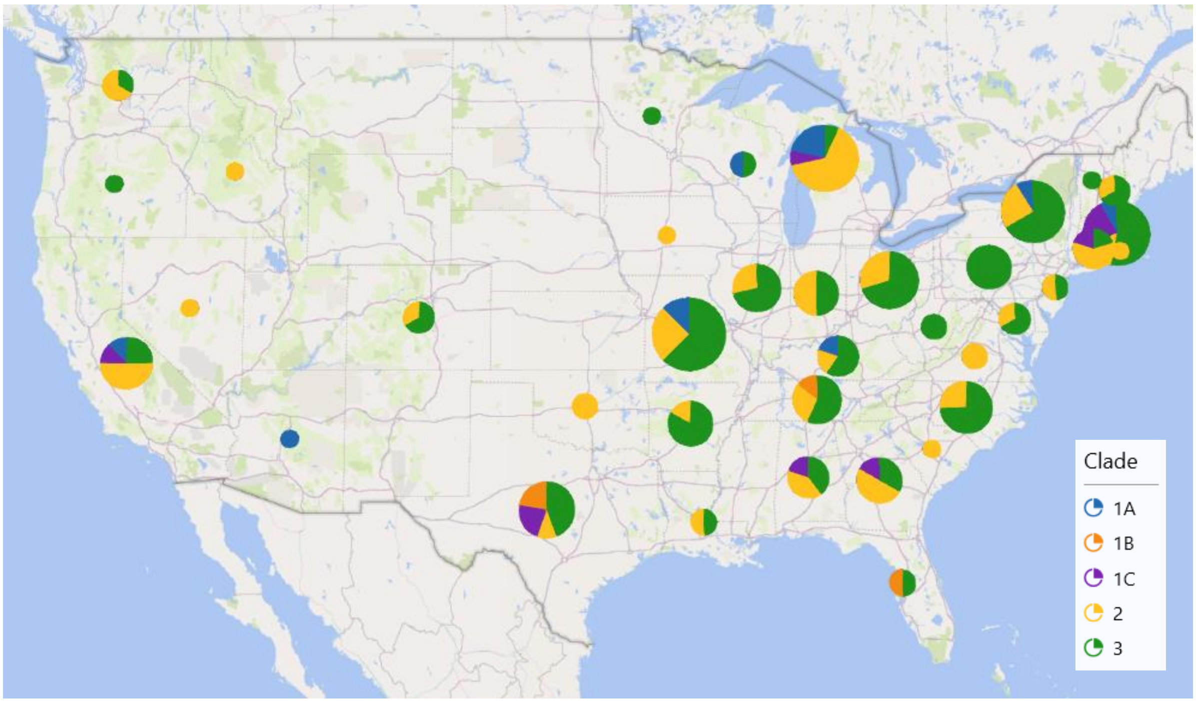

Figure S2. CF infection associated with each *B. gladioli* clade is widely distributed across the United States of America. The state location of the submitting CF treatment centre for 181 CF *B. gladioli* strains in the collection are shown. The *B. gladioli* clade type was plotted against geographic location using the Power Map tool in Microsoft Excel Office 365. The circle size is proportional to the total number of strains at each location and coloured segments indicate the proportion of each clade present (see key).

Figure S3. UHPLC-ESI-Q-TOF-MS analysis of metabolites produced by *B. gladioli* isolates. Structures, calculated masses, extracted ion chromatograms (EICs) and high resolution mass spectra are displayed for each of the following compounds (corresponding strain): (a) toxoflavin (BCC1883); (b) enacyloxin IIa (BCC1883); (c) bongkrekic acid (BCC1710); (d) icosalide A1 (BCC0238); (e) gladiolin (BCC0238); (f) sinapigladioside (BCC1883); and (g) caryoynencin (BCC1883). The MS spectra correspond to the region underneath the peak with an asterisk in each instance.

(a) Toxoflavin

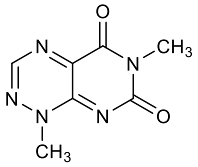

Toxoflavin
Chemical Formula: $C_7H_7N_5O_2$
Exact Mass: 193.0600
$[M+H]^+$: 194.0673
$[M+Na]^+$: 216.0492

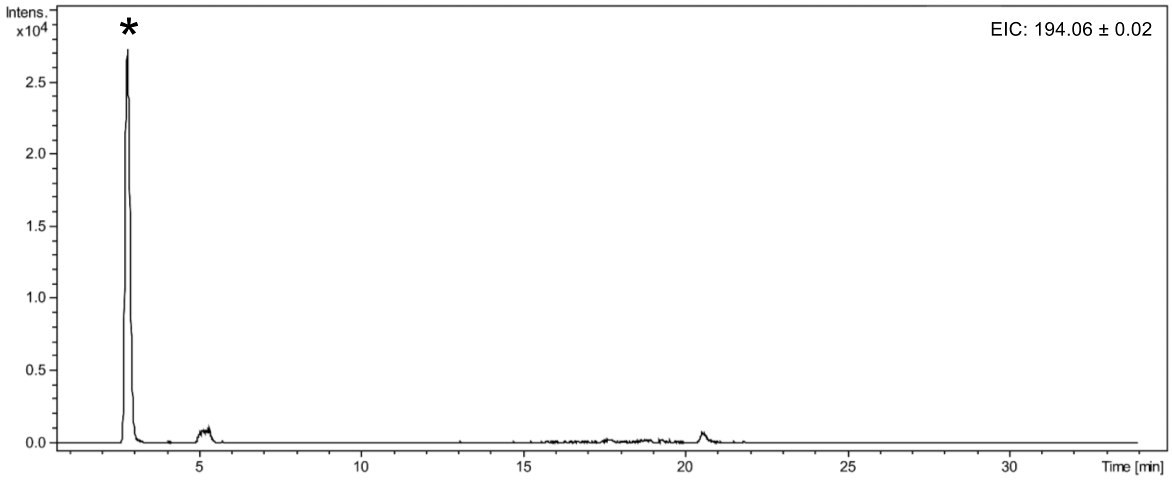

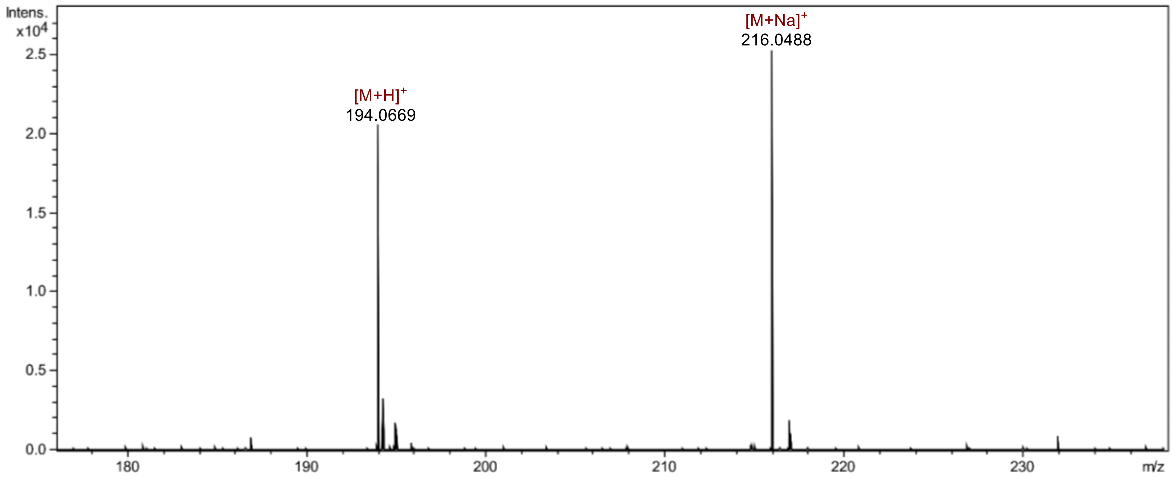

(b) Enacyloxin IIa

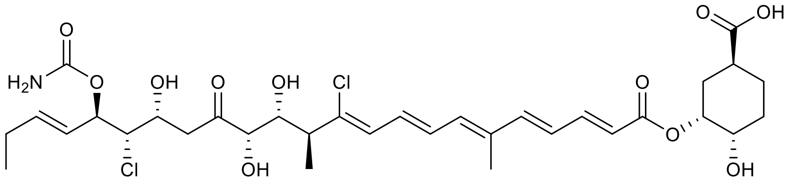

Enacyloxin IIa
Chemical Formula: $C_{33}H_{45}Cl_2NO_{11}$
Exact Mass: 701.2370
$[M+H]^+$: 702.2442
$[M+Na]^+$: 724.2262

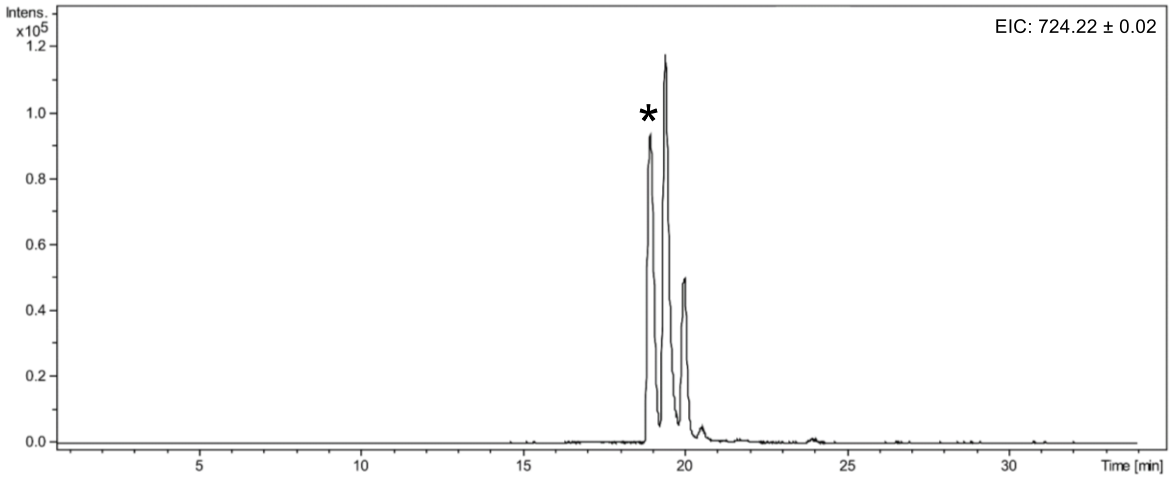

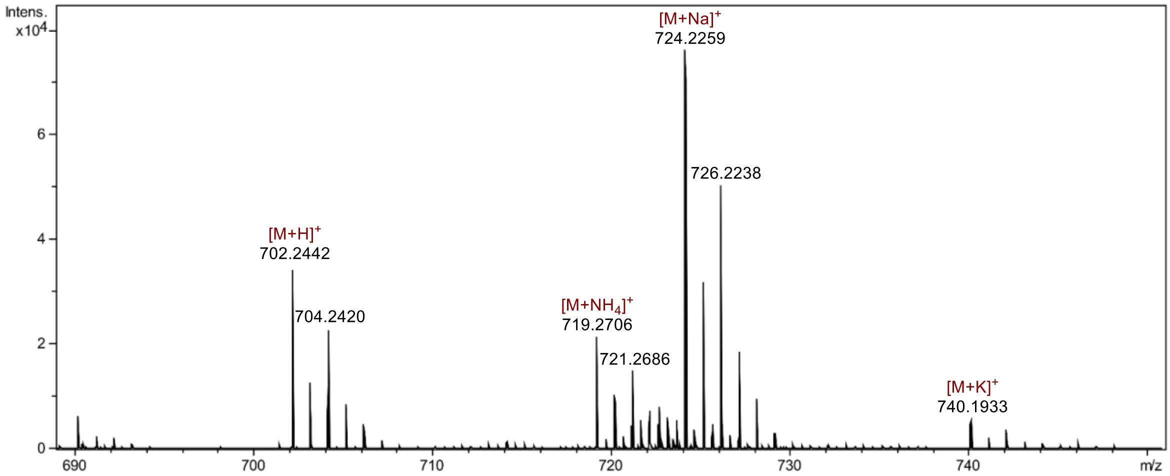

(c) Bongkrekic acid

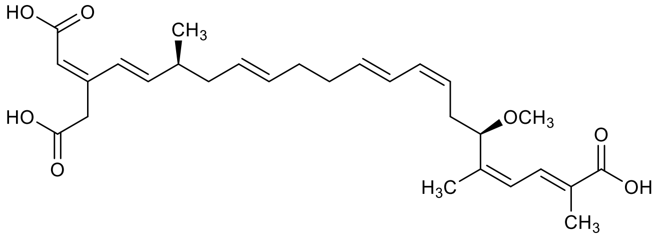

Bongkrekic Acid
Chemical Formula: $C_{28}H_{38}O_7$
Exact Mass: 486.2618
$[M+H]^+$: 487.2690
$[M+Na]^+$: 509.2510

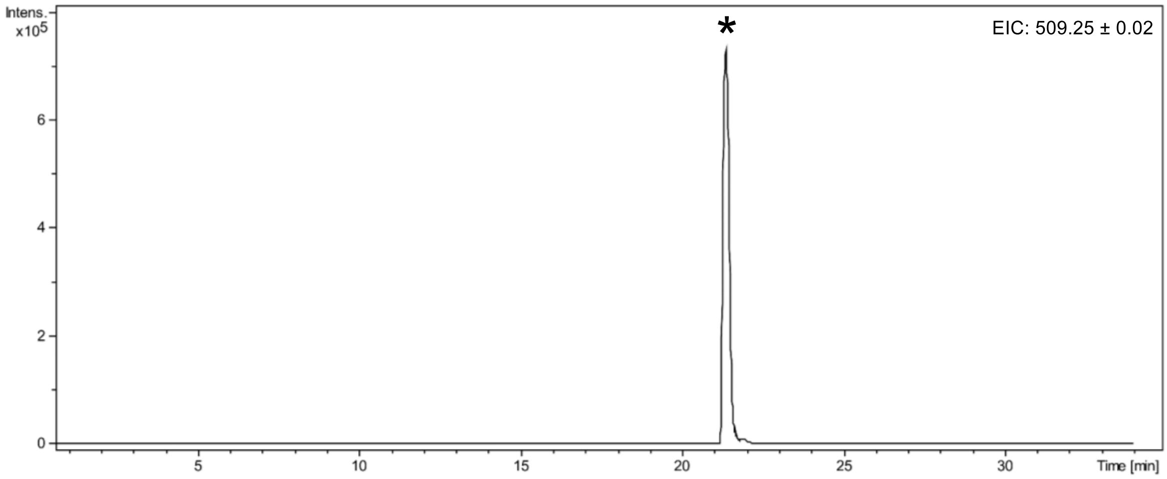

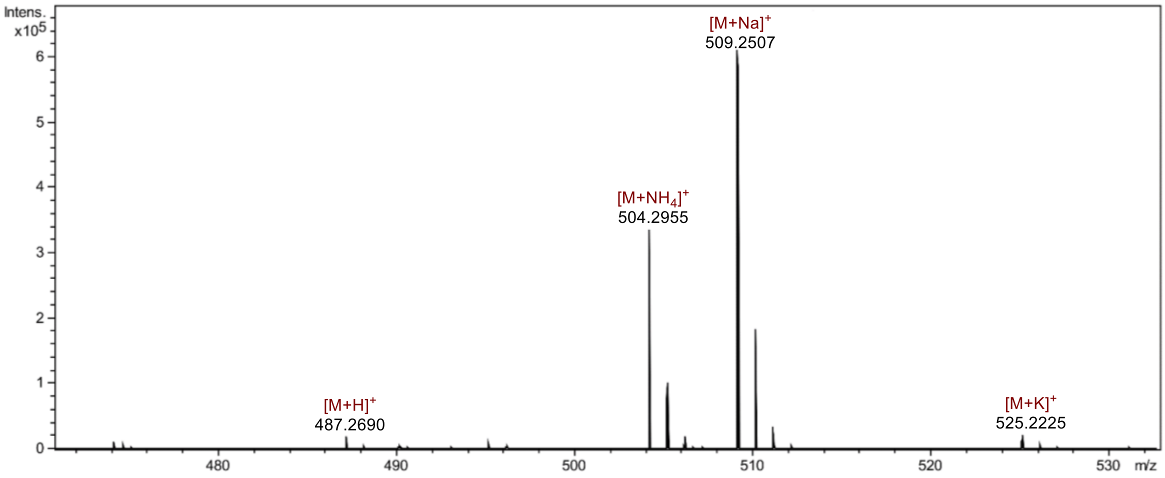

(d) Icosalide A1

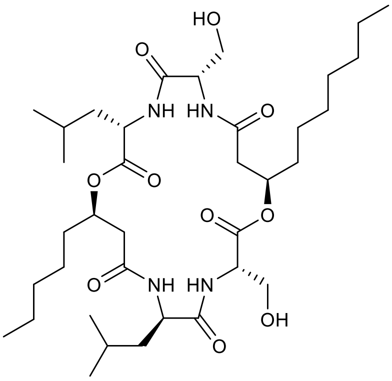

Icosalide A1

Chemical Formula: $C_{36}H_{64}N_4O_{10}$

Exact Mass: 712.4622

$[M+H]^+$: 713.4695

$[M+Na]^+$: 735.4515

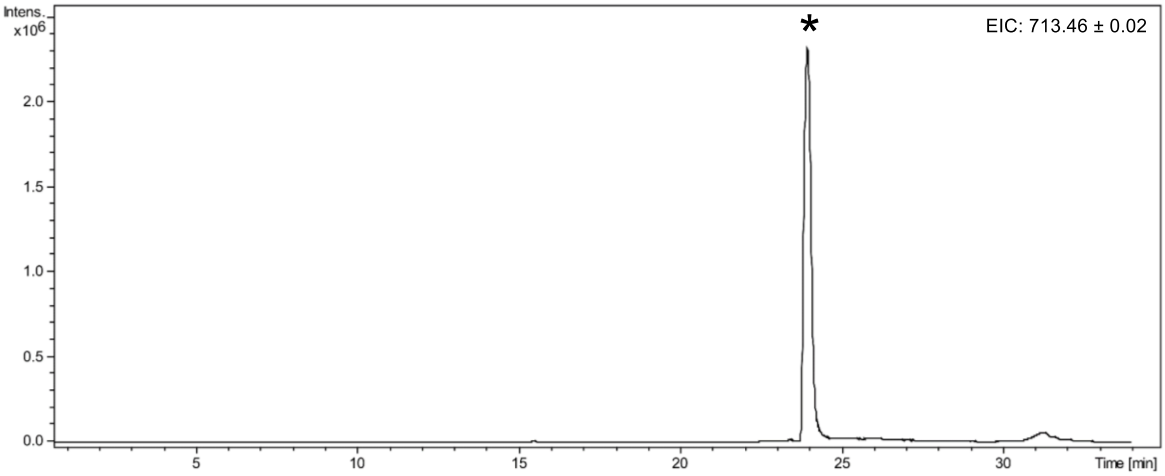

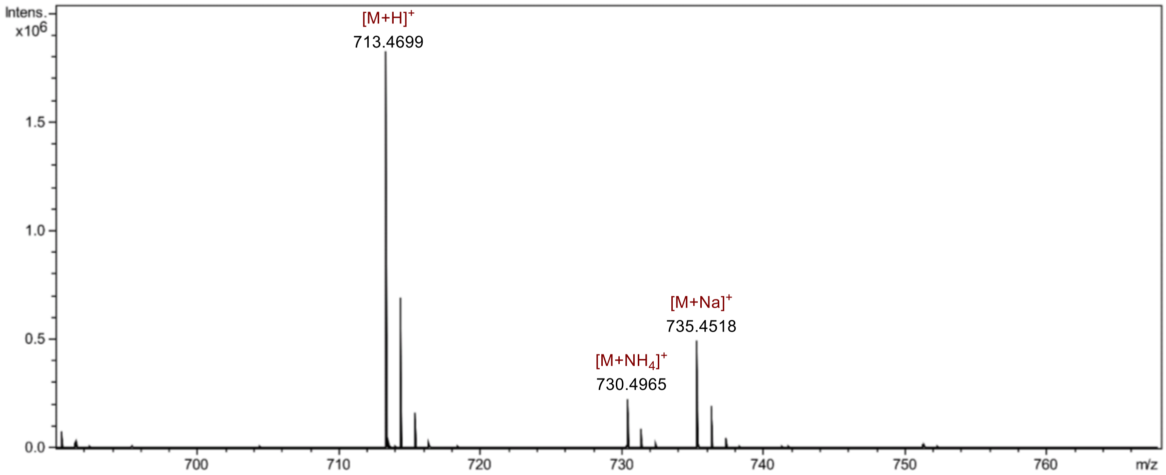

(e) Gladiolin

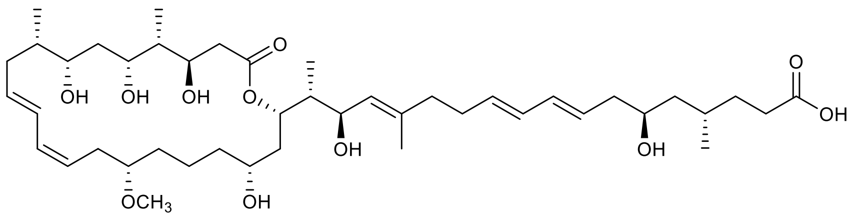

Gladiolin

Chemical Formula: $C_{44}H_{74}O_{11}$

Exact Mass: 778.5231

$[M+H]^+$: 779.5304

$[M+Na]^+$: 801.5123

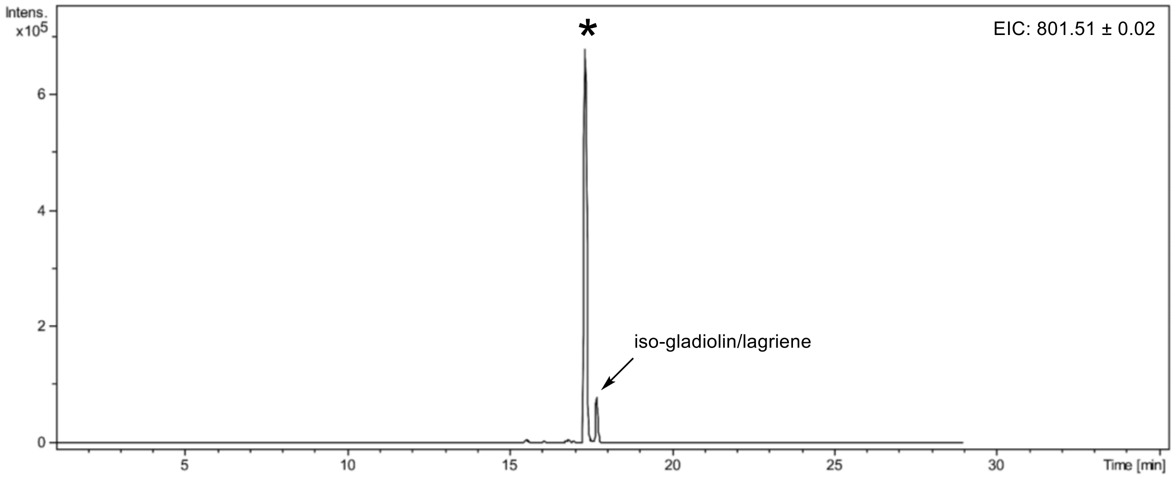

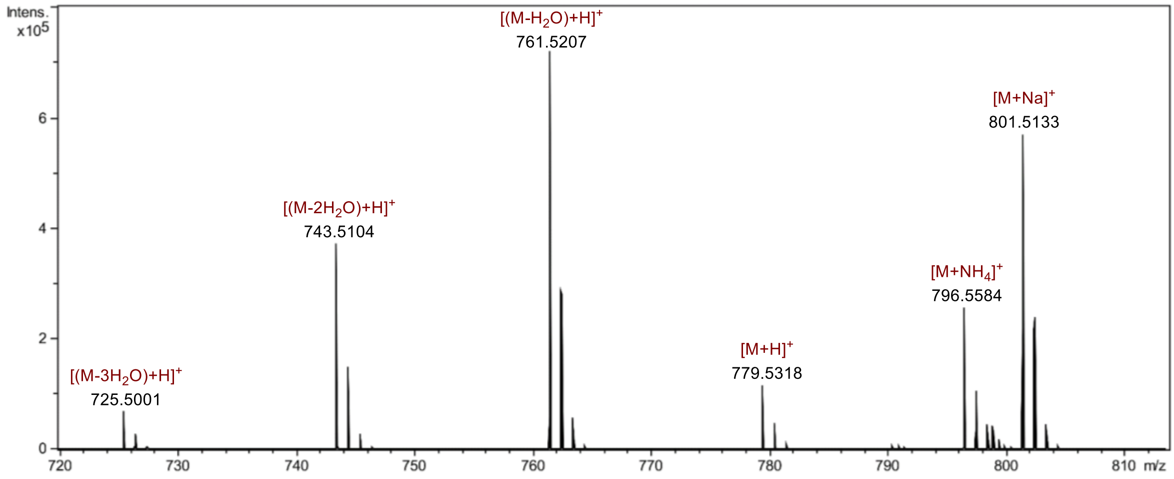

(f) Sinapigladioside

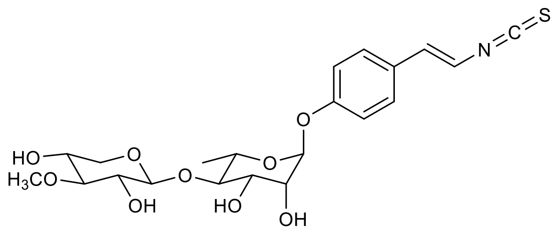

Sinapigladioside
Chemical Formula: $C_{21}H_{27}NO_9S$
Exact Mass: 469.1407
$[M\text{-}H]^-$: 468.1334
$[(M+Na)\text{-}2H]^-$: 490.1153

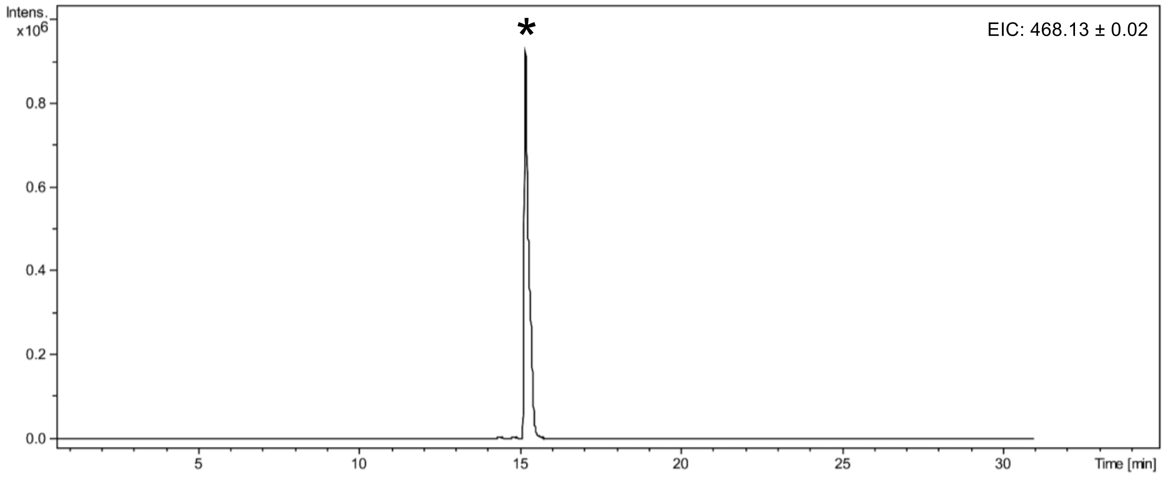

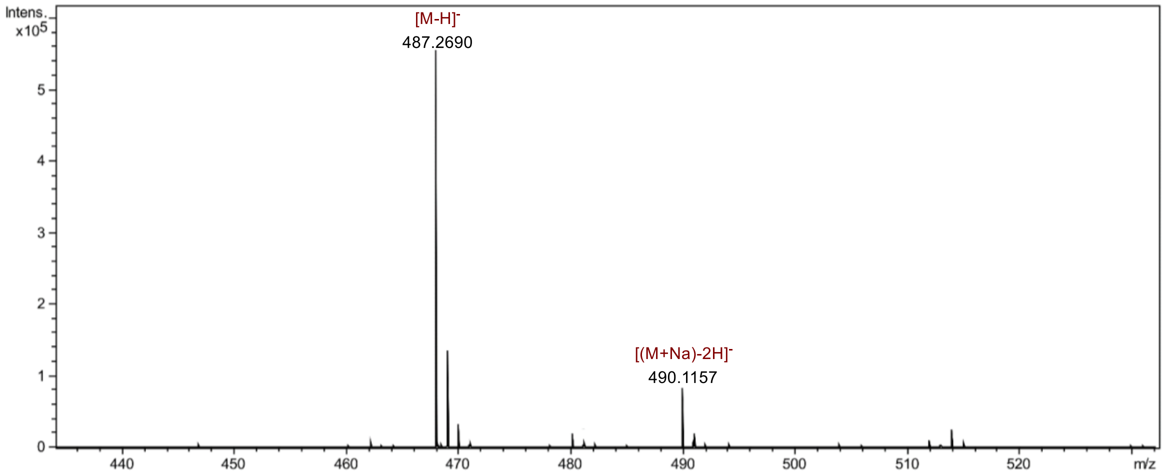

(g) Caryoynencin

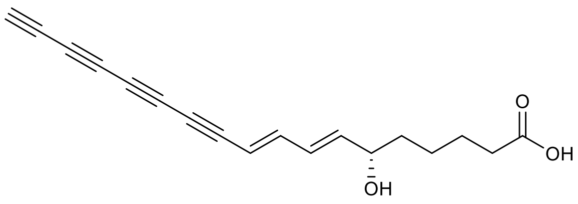

Caryoynencin
Chemical Formula: $C_{18}H_{16}O_3$
Exact Mass: 280.1099
$[M-H]^-$: 279.1027
$[(M+Na)-2H]^-$: 301.0846

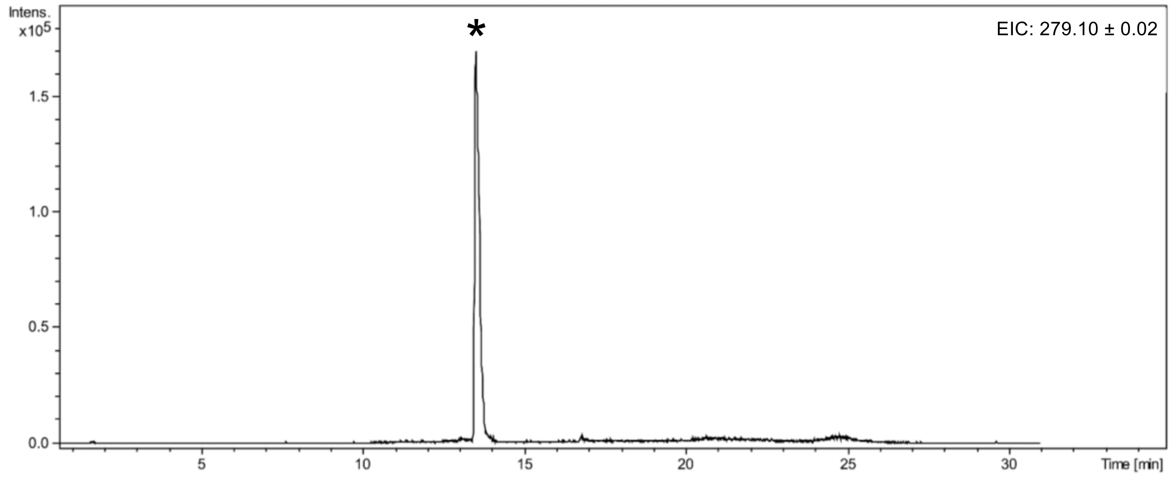

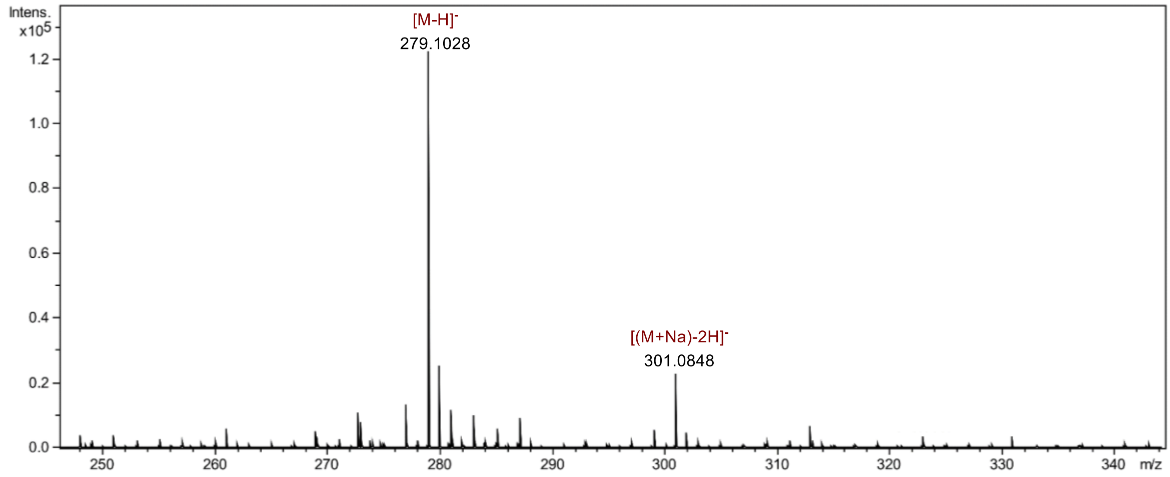

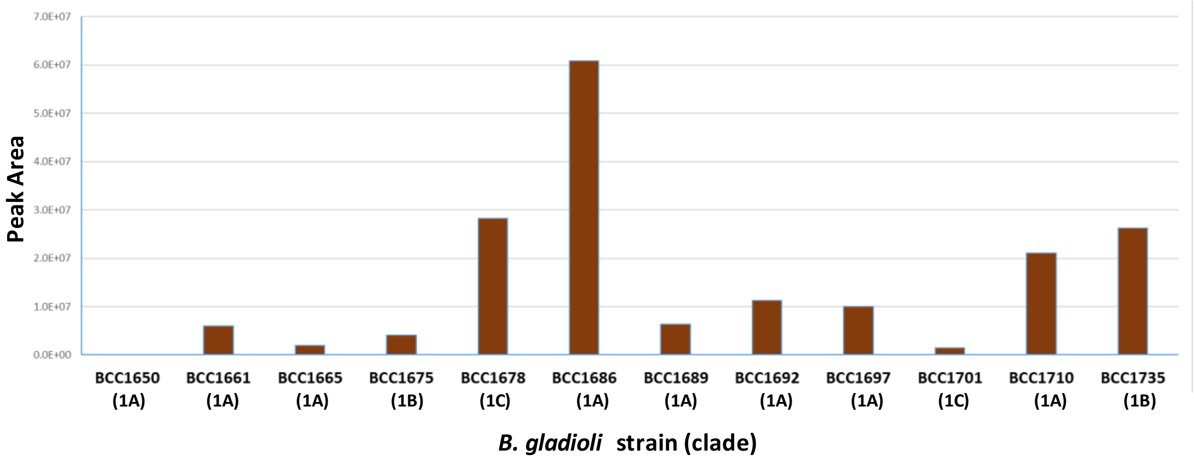

Figure S4. *B. gladioli* clade 1 strains have a variable ability to produce bongkrekic acid. Production of the toxin was evaluated by quantitative HPLC analysis (the mean peak height is plotted) of spent agar extracts analysed after 3 days growth of *B. gladioli* at 37ºC on BSM-G medium. The mean peak height for triplicate extracts is plotted for: 8 clade 1A, 2 clade 1B, and 2 clade 1C strains (isolate names and clade are indicated). Peak height was measured at 210-400 nm.

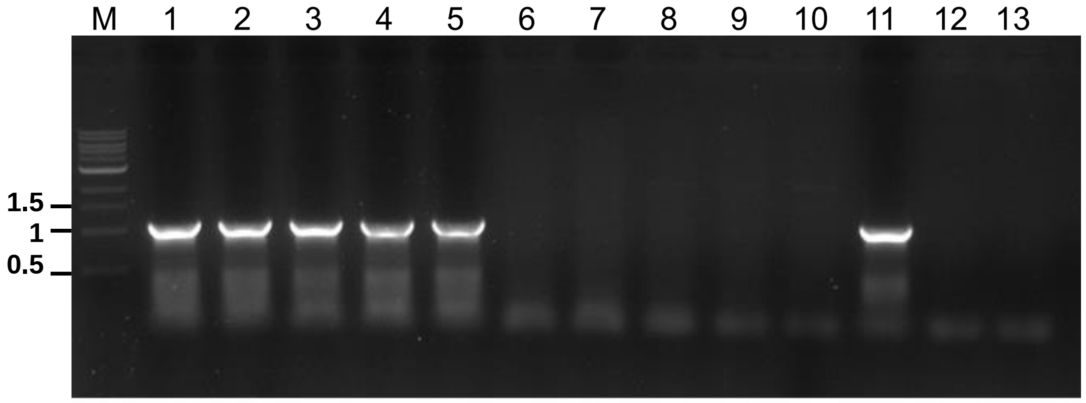

Figure S5. Detection of *B. gladioli* clade 1 strains by bongkrekic acid *bonA* gene PCR. An example PCR to detect the *bonA* gene of toxin positive clade 1 *B. gladioli* strains is shown with lanes as follows: M, molecular size markers (relevant size in kb are shown); 2 to 5 are bongkrekic acid positive strains BCC1710, 1650, 1661, 1735 and 1701, respectively; 6 to 10 are bongkrekic negative strains BCC1622, a colony variant 1622c2, 1723, 1620 and 1662, respectively; 11 is the positive pathovar control strain *B. gladioli* pv. *cocovenenans* LMG 18113; 12 is a negative control strains BCC0238 from clade 3; and lane 13 is the water PCR blank.

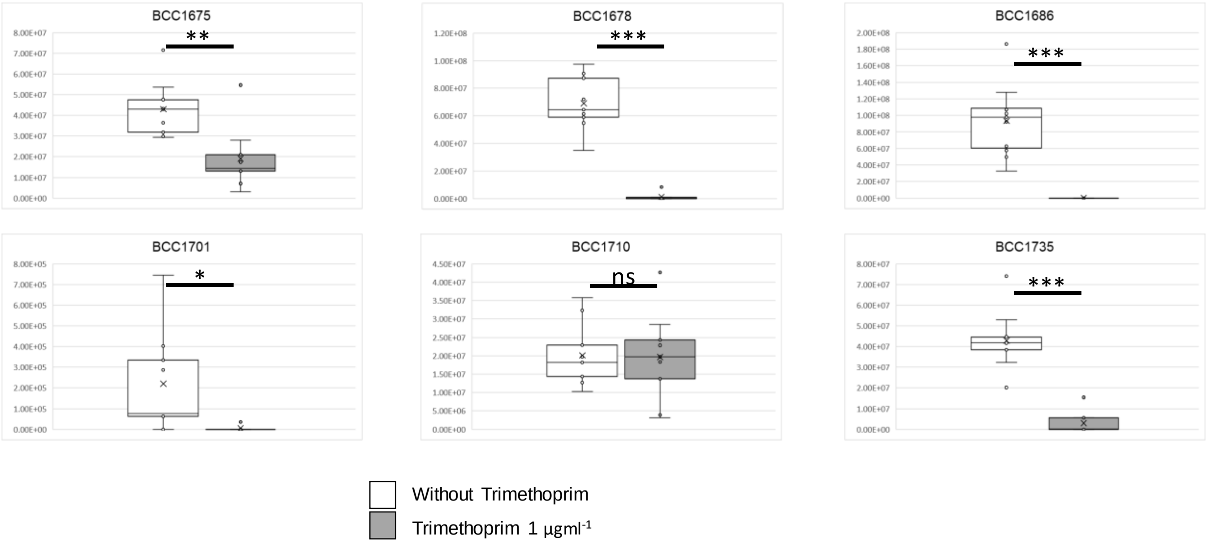

Figure S6. Subinhibitory trimethoprim reduces bongrekic acid production. HPLC analysis of bongkrekic acid production in 6 group 1 *B. gladioli* is shown (clade 1A, BCC1686 and BCC1710; clade 1B, BCC1675 and BCC1735; and clade 1C, BCC1678 and BCC1701). Bongkrekic acid production in the absence (white boxes) and presence (1 µg/mL; grey boxes) of the antibiotic trimethoprim is shown (n=9). Significant suppression (*$p<0.05$); **$p<0.01$; and ***$p<0.001$) of toxin production in the presence of trimethoprim occurred in 5 strains, with non-significant (ns) change observed for strain BCC1710. Peak height measured at 210-400 nm.
